# uPAR‐Targeting T Cell Engager Exerts Senolytic Effects in Mice and Non‐Human Primates With Serum Aminotransferase Activity as a Safety Monitor

**DOI:** 10.1111/acel.70742

**Published:** 2026-09-28

**Authors:** Jian Deng, Xiaozhu Zeng, Jing Guo, Lihong Li, Shirui Zou, Nan Liu, Shuai He, Lanzhen Yan, Qiong Wang, Yongjie Zhu, Ben Wang, Dong Yang, Longbao Lv, Xudong Zhao

**Affiliations:** ^1^ Department of Targeting Therapy & Immunology and Laboratory of Cell Engineering & Immunotherapy, Cancer Center and State Key Laboratory of Respiratory Health and Multimorbidity and National Clinical Research Center for Geriatrics and Frontiers Science Center for Disease‐Related Molecular Network West China Hospital, Sichuan University Chengdu Sichuan China; ^2^ Kunming Institute of Zoology & National Resource Center for Non‐Human Primates Chinese Academy of Sciences Kunming Yunnan China; ^3^ Cancer Institute (Key Laboratory of Cancer Prevention and Intervention, China National Ministry of Education), the Second Affiliated Hospital Zhejiang University School of Medicine Hangzhou China

**Keywords:** senescent cell, senolytics, T cell engager, uPAR

## Abstract

Senolytic CAR‐T cells targeting uPAR induce dose‐dependent toxicities. Although dosage adjustment mitigates these toxicities in laboratory mice, clinical translation remains challenging owing to the unpredictability of CAR‐T cell proliferation across heterogeneous patient populations. In contrast, bispecific T‐cell engagers (BiTEs) offer a safer alternative with superior dose‐titratability. Here, we have developed a GFD‐CD3 BiTE by employing the growth factor‐like domain (GFD) of the natural ligand uPA as the targeting moiety to target uPAR‐positive senescent cells. Dose‐range finding studies revealed that high‐dose GFD‐CD3 predominantly induced hepatotoxicity through T cell–mediated attack on hepatic endothelial cells. Crucially, under transaminase‐guided monitoring, low‐dose GFD‐CD3 effectively eliminated senescent cells and alleviated age‐related pathologies in aged mice and non‐human primates without adverse effects. These findings establish GFD‐CD3 as a clinically translatable senolytic agent and provide the compelling preclinical evidence for BiTE as a senolytic strategy.

## Graphical abstract

GFD‐CD3, a uPAR‐targeting T‐cell engager, effectively cleared senescent cells and mitigated age‐related pathologies with a favorable safety profile under transaminase‐guided monitoring.

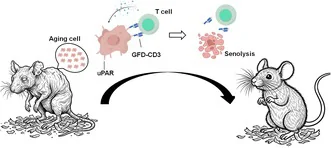

## 1 Introduction

Cellular senescence is a canonical hallmark and driving force of aging. Studies have shown that transplantation of even a small number of senescent cells can promote the aging process in mice (Xu et al. 2017; Xu et al. 2018). Conversely, the elimination of senescent cells has been demonstrated to extend healthspan and improve aging‐related phenotypes (Gasek et al. 2021). Consequently, the development of safe and effective treatments that induce the lysis of senescent cells, termed senolytics, is emerging as a promising anti‐aging strategies (Gasek et al. 2021). A landmark example is the combination of dasatinib (D) and quercetin (Q), which was found in in vitro and in vivo studies to effectively eliminate senescent cells, thereby providing proof‐of‐concept for pharmacological interventions that target aging and age‐related pathologies (Zhu et al. 2015). Subsequent studies have identified additional senolytic candidates, including fisetin and UBX0101 (Chin et al. 2023; Mahoney et al. 2024; Martel et al. 2020), which show potential in ameliorating age‐related diseases (Guarente et al. 2024). Several of these agents are now undergoing active clinical evaluation to assess their efficacy and safety profiles (Guarente et al. 2024; Nambiar et al. 2023).

To achieve senolytic effects with improved safety and efficacy, an alternative approach involves developing highly specific immunotherapies that target membrane markers on senescent cells. For example, senolytic vaccines targeting CD153 or glycoprotein non‐metastatic melanoma protein B (GPNMB) were reported to effectively induce the clearance of senescent cells, thereby alleviating the aging phenotype in mice (Suda et al. 2021; Yoshida et al. 2020). Moreover, CAR T cells targeting membrane markers such as uPAR and NKG2D ligands have been shown to eliminate senescent cells in aged mice (Amor et al. 2024; Amor et al. 2020; Yang et al. 2023). uPAR, which plays crucial roles in wound healing, fibrinolysis and tumor formation through remodeling of the extracellular matrix, was identified as a broadly expressed marker on senescent cells and tumor cells (Rossi and Abdelmohsen 2021; Suryadevara et al. 2024). The uPAR‐targeted CAR T cells effectively eliminated senescent cells in liver fibrosis induced by carbon tetrachloride (CCl4) or diet and alleviated the associated pathologies (Amor et al. 2020). Furthermore, treatment with these CAR‐T cells ameliorated metabolic dysfunction in naturally aged mice by eliminating accumulated senescent cells (Amor et al. 2024). However, while senescent cells were effectively eliminated by low doses of uPAR CAR T cells (0.5 × 10^6^ per mouse), a relatively high dose (2 × 10^6^ of uPAR CAR T cells per mouse) induced hypothermia and weight loss accompanied by a slight increase in serum cytokine levels (Amor et al. 2020). These findings highlight the need for safer uPAR‐targeting senolytics, especially for antiaging purposes. Potential strategies include the use of Bispecific T‐cell engagers (BiTEs), which recruit endogenous T cells to eliminate target cells with enhanced dose‐titratability and reduced toxicity (Subklewe 2021). Another promising approach involves employing natural ligand/receptor interactions as recognition domain in immunotherapy, which is known to result in reasonable safety profiles due to their moderate affinity compared to antibody‐derived single‐chain variable fragments (scFv) (Arcangeli et al. 2017; Wei et al. 2023).

In this study, we identified hepatotoxicity as the primary dose‐limiting toxicity of uPAR‐targeted therapies, stemming from uPAR expression in liver endothelial cells. We further demonstrated that this risk can be effectively managed through the monitoring of serum transaminases. Crucially, low‐dose GFD‐CD3 treatment successfully eliminated senescent cells and reversed age‐related pathologies in both naturally aged mice and non‐human primates, underscoring its potential as a clinically viable senolytic therapy.

## 2 Results

### 2.1 GFD‐CD3 Mediates the Lysis of Human Senescent Cells by T Cells In Vitro

The use of natural receptors or ligands as recognition domains confers moderate affinity to immunotherapy, allowing for the precise targeting of high‐antigen‐expressing cells and minimal damage to healthy tissues (Hanssens et al. 2022; Wei et al. 2023). We constructed a uPAR‐directed BiTE, termed hGFD‐CD3, by sequentially linking an Igκ signal peptide, the GFD domain of human uPA (aa1‐46, a natural uPAR ligand), an anti‐human CD3 scFv, and two non‐functional Fc regions (Figure 1A). The CD3ε scFv sequence is derived from acapatamab, which has been shown to bind to human and monkey CD3 molecules with high affinity and specificity (Deegen et al. 2021). A counterpart construct lacking the hGFD domain served as a negative control (Figure 1A). These constructs were introduced into HEK‐293F cells, and the resultant expressed proteins were purified from culture medium (Figure S1A,B). First, we tested the ability of hGFD‐CD3 to bind to human uPAR on senescent cells and CD3 on T cells. Senescent cells were induced in human fetal lung fibroblasts HEL1 via P16^INK4a^ overexpression or etoposide treatment, as previously described (Yang et al. 2023), with successful induction confirmed by SA‐β‐Gal staining (Figure S2A). As expected, uPAR expression was specifically detected in senescent cells but not in their counterparts (Figure S2B). Flow cytometry demonstrated effective binding of hGFD‐CD3 to uPAR‐positive senescent cells and CD3‐positive T cells (Figure 1B,C). Immunofluorescence studies further confirmed the accumulation of BiTE around treated cells (Figure S2C,D). Next, we cocultured senescent cells with T cells at different concentrations of the engager and found that senescent cells were killed in a dose‐dependent manner, with an IC50 value as low as 0.015 nM, which was accompanied by an increase in cytokine levels, such as for TNF‐α and IFN‐γ (Figure 1D,E and Videos S1 and S2). No obvious cytotoxicity was detected in young cells or senescent cells treated with the control engager (Figure 1D, Figure S3). Moreover, hGFD‐CD3 treatment significantly upregulated the T‐cell activation markers CD69 and CD25 (Figure 1F), as well as the release of granzyme (Figure 1E), in a co‐culture system of senescent and T cells. These results demonstrate that hGFD‐CD3 can effectively bind to senescent cells and T cells and mediate the elimination of senescent cells by T cells.

**FIGURE 1:**
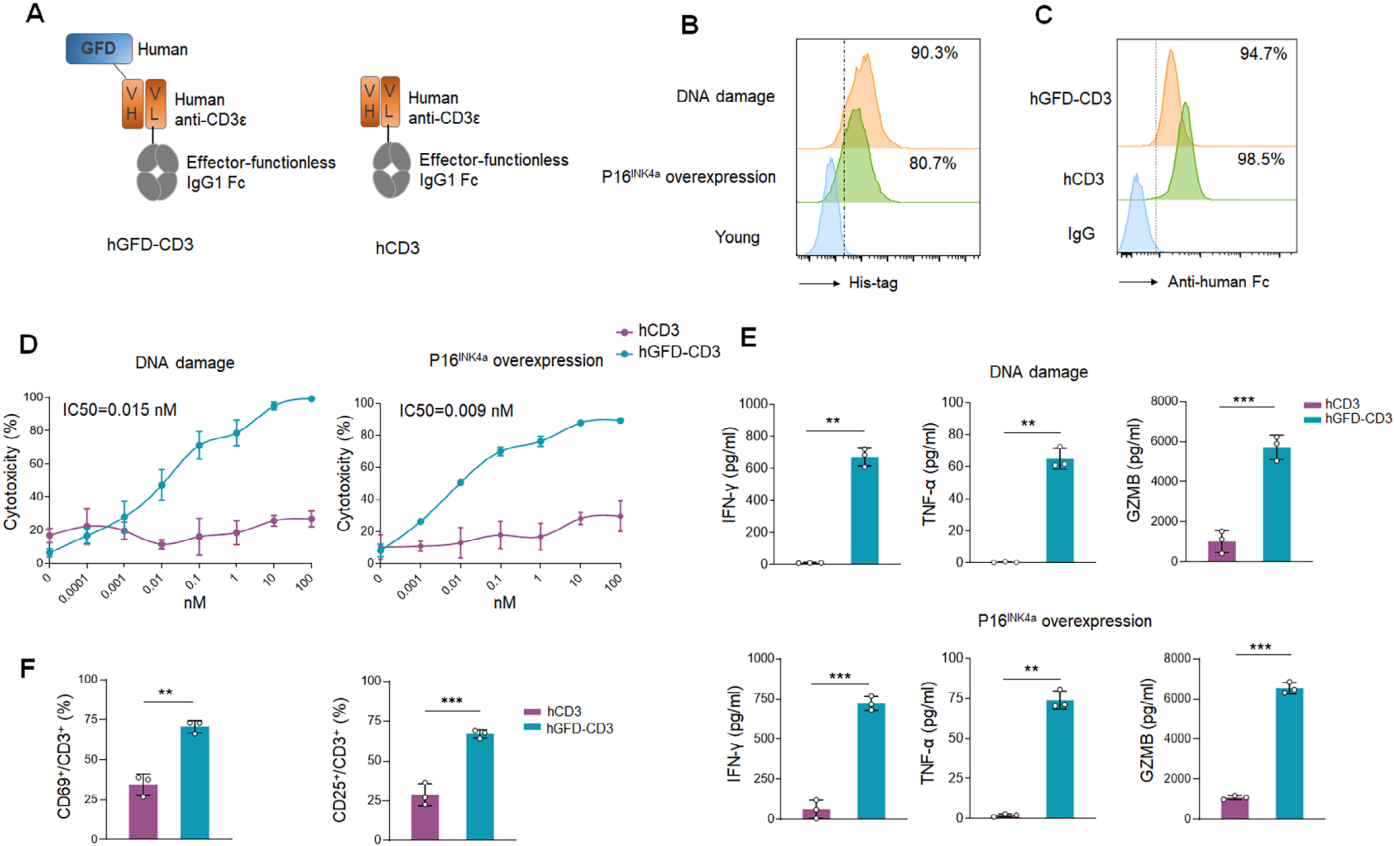
hGFD‐CD3 mediates the clearance of senescent cells by T cells in vitro. (A) A schematic diagram of hGFD‐CD3 and the negative control used in this study. (B) Semiquantitative analysis of the cell surface binding of hGFD‐CD3 to young uPAR‐HEL1 cells and uPAR+ HEL1 senescent cells via flow cytometry. (C) Semiquantitative analysis of the cell surface binding of hGFD‐CD3 and hCD3 to human CD3+ T cells via flow cytometry. (D) Dose–response study of hGFD‐CD3 mediated T cell cytotoxicity against senescent cells (E: T = 1: 1). Each data point represents the mean of a biological replicate (*n* = 2). (E) Cytokine release studies using ELISA from cell supernatants in experiments where 100 ng/mL hGFD‐CD3 was applied. (F) T cell activation was assessed by measuring CD25 and CD69 levels on CD3+ T cells by flow cytometry. All experiments were performed in biological triplicate. Data are presented as mean ± SD of triplicate samples and analyzed by two‐tailed unpaired Student's *t*‐test with Welch's correction (e, f). **p ≤ 0.05; **p ≤ 0.01; ns, not significant.

### 2.2 High‐Dose GFD‐CD3 Induces Hepatotoxicity by Targeting Hepatic Endothelial Cells

Building on our in vitro findings, we next assessed the in vivo effects of GFD‐CD3. First, we engineered a mouse‐specific GFD‐CD3 BiTE (mGFD‐CD3) containing the mouse uPA GFD domain and an anti‐mouse CD3 scFv fragment (Figures S1C and S4A). A parallel construct devoid of the mGFD domain served as a negative control. To evaluate mGFD‐CD3 BiTE functionality, we first induced senescence in primary mouse embryonic fibroblasts (MEFs) from C57BL/6 mice using either etoposide or replicative exhaustion, which was confirmed by enhanced SA‐β‐Gal activity (Figure S4B). Flow cytometry analysis confirmed that mGFD‐CD3 efficiently bound to senescent, but not young, MEFs (Figure S4C). The CD3ε scFv moiety of the BiTE also showed efficient and specific binding to mouse T cells (Figure S4D). In a co‐culture system of mouse T cells and senescent MEFs, the addition of mGFD‐CD3 mediated potent, dose‐dependent T cell cytotoxicity against the target cells (Figure S4E).

To explore mGFD‐CD3 dosages in aged mice, we treated 22‐month‐old mice with a dose series of mGFD‐CD3 (0.01, 0.03, 0.1, 0.3, and 1 mg/kg). Treatment at 1 mg/kg concentration of this BiTE led to mouse death within 24 h, while a dose of 0.3 mg/kg treatment resulted in adverse features including ruffled fur, low spirits, and anorexia, accompanied by a 20‐ to 30‐fold increase in serum AST and ALT levels in the serum and a 4‐fold increase in IL‐6 levels (Figure S4F,G). Histopathological analysis revealed hepatic damage but no pathologically evident alterations in other organs examined (Figure 2A, Figure S4H). At doses below 0.3 mg/kg, no obvious abnormalities were observed except for a modest elevation in serum IL‐6 (Figure S4G). These results demonstrate that high doses of mGFD‐CD3 induce hepatotoxicity and lethality in treated aged mice.

**FIGURE 2:**
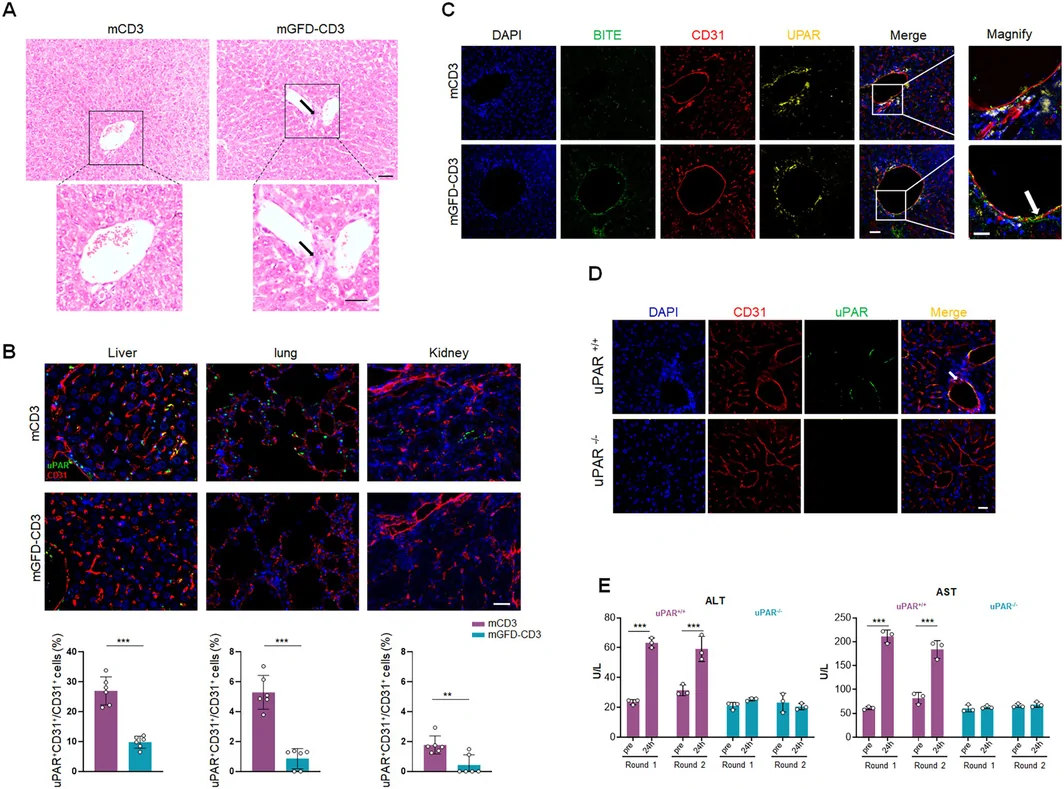
mGFD‐CD3 induces hepatotoxicity by targeting hepatic endothelial cells in mice. (A) H&E analysis performed at 24 h post‐injection of BiTE in the liver tissues of naturally aged mice. Scale bar, 30 μm (original) and 15 μm (magnified). (B) Immunofluorescence analysis was conducted on liver, lung, and kidney samples from naturally aged mice collected 24 h post‐BiTE injection (*n* = 6 view field per each treatment group). Scale bar, 30 μm. (C) Immunofluorescence analysis of liver samples of naturally aged mice taken 4 h post‐injection of BiTE to examine the colocalization of BiTE, CD31, and uPAR. Scale bar, 30 μm (original) and 15 μm (magnified). (D) Immunofluorescence analysis to examine the colocalization of CD31 and uPAR in the liver tissues of wild type mice (WT) and uPAR knockout mice; (*n* = 3 mice per treatment group). Scale bar, 30 μm. (E) Levels of AST and ALT in serum samples from mice before and after BiTE injection were determined with a Biochemical Analyzer; (*n* = 3 mice per treatment group). Data is presented as mean ± SD and analyzed by unpaired two‐tailed Student's *t*‐test with Welch's correction. ***p ≤ 0.001.

Available single‐cell RNA sequencing data reveals that in most tissues, uPAR‐positive cells are predominantly hematopoietic, including macrophages, monocytes, and B cells. Notably, liver endothelial cells are among the very few non‐hematopoietic cell types that express uPAR (Amor et al. 2024). Given that damage to liver endothelial cells is a well‐established mechanism of liver damage, we hypothesized that uPAR expression on these cells renders them targets for T cell‐mediated lysis by mGFD‐CD3, thereby exacerbating hepatic injury. To investigate this possibility, we first confirmed uPAR expression in liver endothelial cells through immunostaining studies. As shown, co‐localization of CD31 and uPAR signals was evident, while co‐localization in lung and kidney cells was rare (Figure 2B). Next, we performed ELISA analysis and discovered that mGFD‐CD3 was present in various tissues, with the highest levels in the liver 24 h after injection, while control protein mCD3 accumulated predominantly in the spleen (Figure S5A). mGFD‐CD3 strongly colocalized with both uPAR and CD31 signals on liver sections as demonstrated by immunofluorescence (Figure 2C). In contrast, mCD3 showed negligible colocalization with these markers (Figure 2C). In lung tissue, mGFD‐CD3 colocalized with uPAR but weakly co‐localized with CD31 (Figure S5B). Consistent with these findings, BiTE treatment led to a significant reduction in CD31‐positive cells (Figure 2B). These data collectively indicate that mGFD‐CD3 induces liver damage by targeting uPAR‐positive liver endothelial cells.

Given that uPAR expression in young liver is low compared to the liver of aged mice, we next investigated whether high‐dose mGFD‐CD3 also targets liver endothelial cells in young mice. Consistent with observations in aged mice, treatment of young mice with mGFD‐CD3 also led to substantial accumulation of the BiTE in the liver and elevated transaminase levels (Figure S6). To further validate whether the observed hepatotoxicity was uPAR‐dependent, we treated uPAR knockout mice with mGFD‐CD3. Consistent with earlier findings, wild‐type uPAR mice showed clear co‐localization of uPAR in CD31‐positive cells, accompanied by significant vascular disruption (Figure 2D, Figure S7A) and liver injury (Figure S7B,C). In contrast, CD31 staining indicated intact hepatic vascular endothelium and normal liver histology in uPAR knockout mice (Figure 2D, Figure S7B). Moreover, administration of mGFD‐CD3 increased serum transaminase levels within 24 h in wild‐type but not uPAR‐knockout young mice (Figure 2E). This effect was reproduced by a second treatment 1 week later (Figure 2E). These results demonstrate that liver injury from mGFD‐CD3 treatment is specifically dependent on uPAR expression by liver endothelial cells. Next, the effects of GFD‐CD3 were evaluated in male mice. Treatment at 0.3 mg/kg significantly elevated serum AST and ALT levels (Figure S7D), with histopathological analysis confirming liver injury; no obvious pathological changes were detected in other organs (Figure S7E). In contrast, the 0.1 mg/kg dose caused no observable abnormalities in any examined tissues (Figure S7E). These findings mirror those in female mice, indicating that GFD‐CD3–induced liver injury is sex‐independent.

Previous studies have speculated that the side effects of high dose uPAR CAR T therapy may be linked to the local accumulation and activation of macrophages (Amor et al. 2020), the precise mechanism remains unclear. Therefore, we next investigated whether uPAR‐targeted therapy would cause hepatotoxicity in macrophage‐deficient NSG mice. A uPAR‐directed CAR was constructed based on the GFD domain of human uPA (Figure S8A). It demonstrated potent and specific cytotoxicity against uPAR‐positive cancer cells and senescent cells (Figure S8B–E). These CAR‐T cells also mediated substantial killing of mouse uPAR‐overexpressing HEK‐293T cells (Figure S8F–H), as human uPA was reported to cross‐bind mouse uPAR (Lin et al. 2010). Intravenous administration of GFD‐CAR‐T cells to tumor‐bearing NSG mice significantly suppressed xenograft growth (Figure S8I). However, the mice gradually developed signs of illness and died. Histopathological examinations revealed extensive necrosis of the liver but no obvious pathological changes in other tested organs (Figure S8J). Notably, there was a dramatic increase in the levels of serum transaminases ALT and AST (Figure S8K). In line with the findings in BiTE‐treated mice, CAR‐T‐treated mice exhibited a significant reduction in CD31 and uPAR expression, accompanied by disruption of vascular architecture (Figure S8L). Collectively, these results demonstrate that uPAR targeting with either GFD‐CAR T cells or GFD‐CD3 can induce hepatotoxicity by targeting hepatic endothelial cells.

### 2.3 mGFD‐CD3 Effectively Mediated the Elimination of Senescent Cells by T Cells in Naturally Aged Mice

Since we found that a 0.1 mg/kg dosage of mGFD‐CD3 did not significantly elevate serum transaminase levels in mice, we asked whether this dosage would be sufficient to eliminate senescent cells in naturally aged mice. The half‐life of the BiTE in the bloodstream was approximately 48 h, however its levels were undetectable after approximately 1 week after injection (Figure 3A). Accordingly, the mice were dosed once each week. After three doses, we examined all major organs, and no significant tissue damage was detected (Figure S9A). Il‐6 and TNF‐α levels were significantly increased approximately 2‐fold compared to control mice, and those levels were well below the threshold that would define a cytokine storm and, furthermore, these levels returned to baseline following the cessation of treatment (Figure S9B,C). Treatment with mGFD‐CD3 significantly reduced the expression of muPAR, P16^INK4a^ and P21 in various tissues (Figure 3B). Additionally, decreased SA‐β‐gal staining intensity was observed in adipose tissue, liver, and lung tissue, further indicating a decline in the number of senescent cells following treatment (Figure 3C,D). Consistent with these findings, RNA‐seq analysis of liver tissue, an organ which has been shown in previous studies to be highly sensitive to uPAR‐targeted antiaging therapies, demonstrated a significant reduction in the expression of SASP genes and enrichment of senescence‐associated genes (SenMayo gene set) (Saul et al. 2022) (Figure 3E–G). The expression of macrophage, T cell, and NK cell markers were all decreased (Figure S9D), accompanied by a significant enrichment of differentially expressed genes in inflammation‐related pathways (Figure S9E), suggesting a reduction in inflammation levels. However, no significant improvements in physical function or muscle fiber density were observed (Figure S10A,B), suggesting such benefits may require a longer intervention period.

**FIGURE 3:**
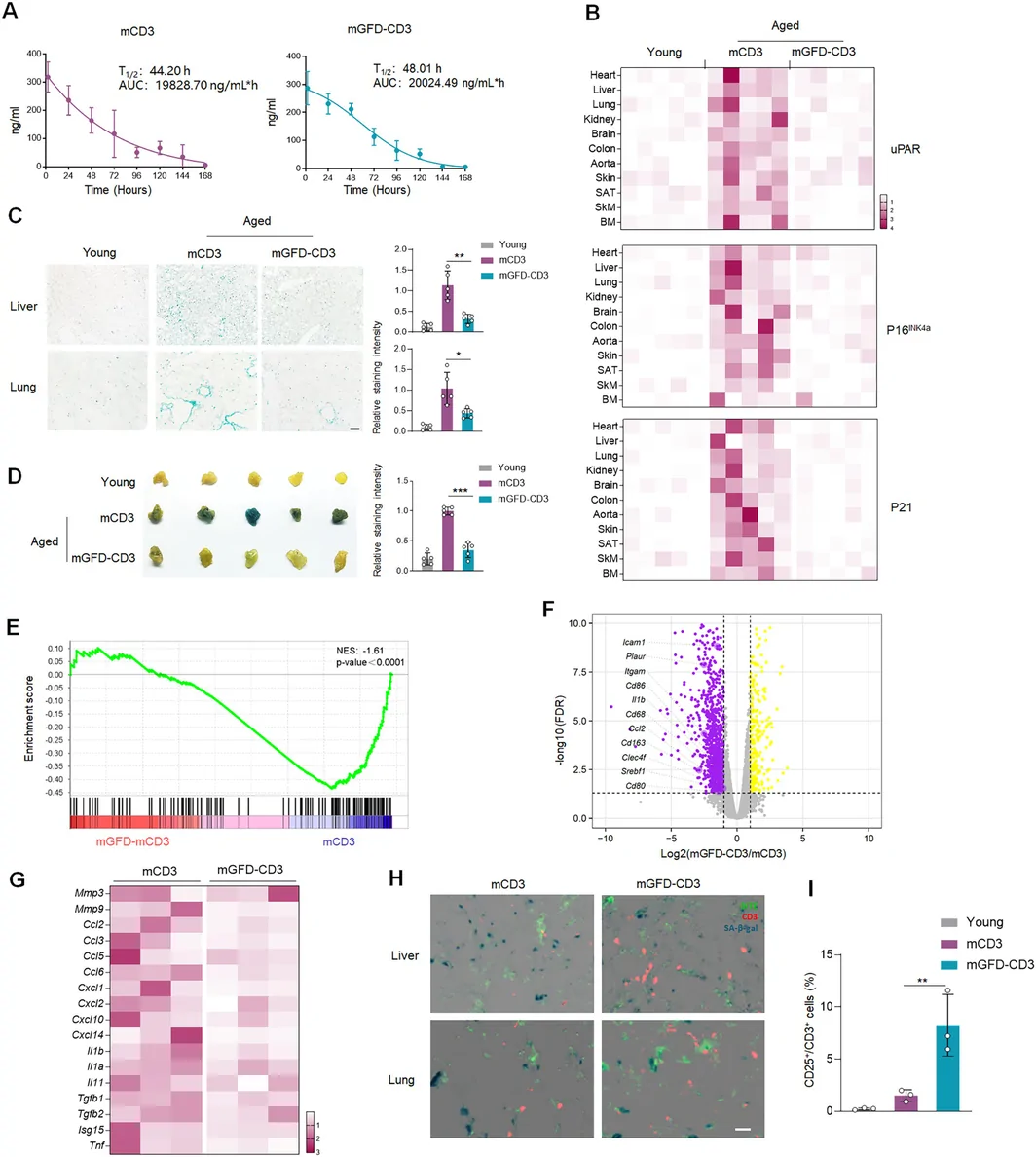
mGFD‐CD3 safely and effectively mediates T‐cell clearance of senescent cells in naturally aged mice. (A) The half‐lives of mGFD‐CD3 and mCD3 negative control in naturally aged mice were analyzed by ELISA. Each data point represents the mean of a biological replicate (*n* = 2). (B) The mRNA expression levels for mouse uPAR, P16^INK4a^, and P21 were detected by qPCR analysis from the indicated tissues from mice treated with mCD3 or mGFD‐CD3 on day 21 (*n* = 5 mice per group). (C) Representative images of SA‐β‐Gal staining of liver and lung tissue samples collected from naturally aged mice treated with mCD3 or mGFD‐CD3 (*left*), with quantification of average staining intensities (ImageJ) (*right*) (*n* = 5). Scale bar, 100 μm. (D) Images of subcutaneous adipose tissue (SAT) after SA‐β‐Gal staining (*left*) and quantification of the average staining intensities (ImageJ) (*right*). SAT was collected from mice treated with mCD3 or mGFD‐CD3 on day 21 (*n* = 5 individual samples per condition). Results from mouse experiments are representative of two independent experiments. (E) Analysis of Senmayo enrichment dataset of liver tissue samples from naturally aged mice treated with mGFD‐CD3 or mCD3 as negative control (*n* = 3 mice per condition). (F) Gene expression volcano plot from the analysis of liver tissue samples collected from mice treated with mCD3 or mGFD‐CD3 (*n* = 3 per condition). (G) Hierarchical clustering heatmap of the senescence‐associated secretory phenotype (SASP) profiles of liver samples (*n* = 3 per condition). (H) Representative images of BiTEs (green), CD3 (red), and SA‐β‐Gal staining (blue green) for liver and lung samples collected from mice treated with BiTEs 24 h post‐injection. (I) Flow cytometry results of CD3^+^ T cells analyzed for CD25 expression after administration of mCD3 or mGFD‐CD3 (*n* = 3 mice per condition). Data is presented as mean ± SD and analyzed by one‐way ANOVA followed by Tukey's multiple comparisons test (C, D, I). *p ≤ 0.05; **p ≤ 0.01; ***p ≤ 0.001; ns, not significant.

To assess the bridging effects of the BiTE between T cells and senescent cells, liver and lung tissues were collected 24 h after treatment for staining analysis. Significant colocalization of SA‐β‐gal, CD3, and mGFD‐CD3 fluorescence was observed, indicating that the BiTE effectively recruited T cells to senescent cells (Figure 3H). Consistently, treatment with mGFD‐CD3 induced T cell activation, as evidenced by an increase in peripheral CD25^+^ T cells in treated mice (Figure 3I). According to available single‐cell sequencing data, macrophages constitute a major senescent cell type across multiple tissues. Pronounced co‐localization of uPAR with the macrophage marker F4/80 was observed in liver and lung tissues. Notably, the abundance of these uPAR‐positive macrophages was significantly reduced after mGFD‐CD3 treatment (Figure S10C). These findings indicate that mGFD‐CD3 effectively reduces the burden of senescent cells by redirecting T cells.

Next, we examined the effects of BiTE treatment on immune cells in peripheral blood. The numbers of neutrophil cells which are known to constitutively express uPAR decreased 6 h after mGFD‐CD3 treatment and returned to baseline within 24 h (Figure S10D). This may be explained by a rapid turnover of neutrophils. A decrease in lymphocytes was also noted at 6 and 24 h after mGFD‐CD3 treatment (Figure S10D). This phenomenon was also observed in other BiTEs and may be attributed to lymphocyte migration into tissues (Bargou et al. 2008; Klinger et al. 2012). Nevertheless, together, these results demonstrate the safety and efficacy of mGFD‐CD3 treatment to eliminate senescent cells.

### 2.4 Elimination of Senescent Cells With mGFD‐CD3 Improves Age‐Related Phenotypes in Mice

To investigate the long‐term impacts of treatment with this engager on the physiological effects of mice, we treated 20‐month‐old mice with mGFD‐CD3 once weekly for 2 weeks, followed by monthly administration. Three months after the initial administration, blood and major tissues were collected for further analysis. Levels of circulating SASP factors such as TNF‐α and IL‐1β in mGFD‐CD3‐treated mice were significantly decreased, indicating an alleviation of systemic inflammation (Figure 4A). Moreover, physiological changes in multiple tissues compared to control‐treated mice suggested improvements to aging‐related phenotypes, as follows. A significant increase in inguinal adipocyte area was observed in mGFD‐CD3‐treated mice compared to control (Figure 4B). Masson's trichrome staining revealed a reduction in positively stained areas within the liver and lung, suggesting that mGFD‐CD3 treatment ameliorated fibrosis in these organs (Figure 4C). Treatment with BiTE also attenuated glomerulosclerosis, as evidenced by PAS staining (Figure 4D). Furthermore, in femoral cancellous bone, TRAP staining demonstrated a slight decrease in osteoclast numbers following mGFD‐CD3 treatment, while Masson's trichrome staining highlighted enhanced osteoblast activity (Figure 4E), an effect consistent with previously reported outcomes for senolytic agents (Farr et al. 2017; Yao et al. 2020). These results indicated that clearance of senescent cells from the bone microenvironment via mGFD‐CD3 may be protective against age‐related bone loss by simultaneously promoting osteoblast function and suppressing osteoclast activity. Next, H&E staining was performed on major tissues and revealed no significant morphological abnormalities in any treatment group (Figure S11A). Serum ALT and AST levels were also found to lie within normal ranges (Figure S11B). Furthermore, administration of mGFD‐CD3 did not adversely affect food intake and body weight in the aged mice (Figure S11C,D). Increase in movement duration and maximum acceleration in open field test in 3 months after mGFD‐CD3 treatment suggested improved motor function (Figure S11E). Taken together, mGFD‐CD3, at the optimized dosage, was found to safely and effectively mitigate age‐related pathologies in naturally aged mice.

**FIGURE 4:**
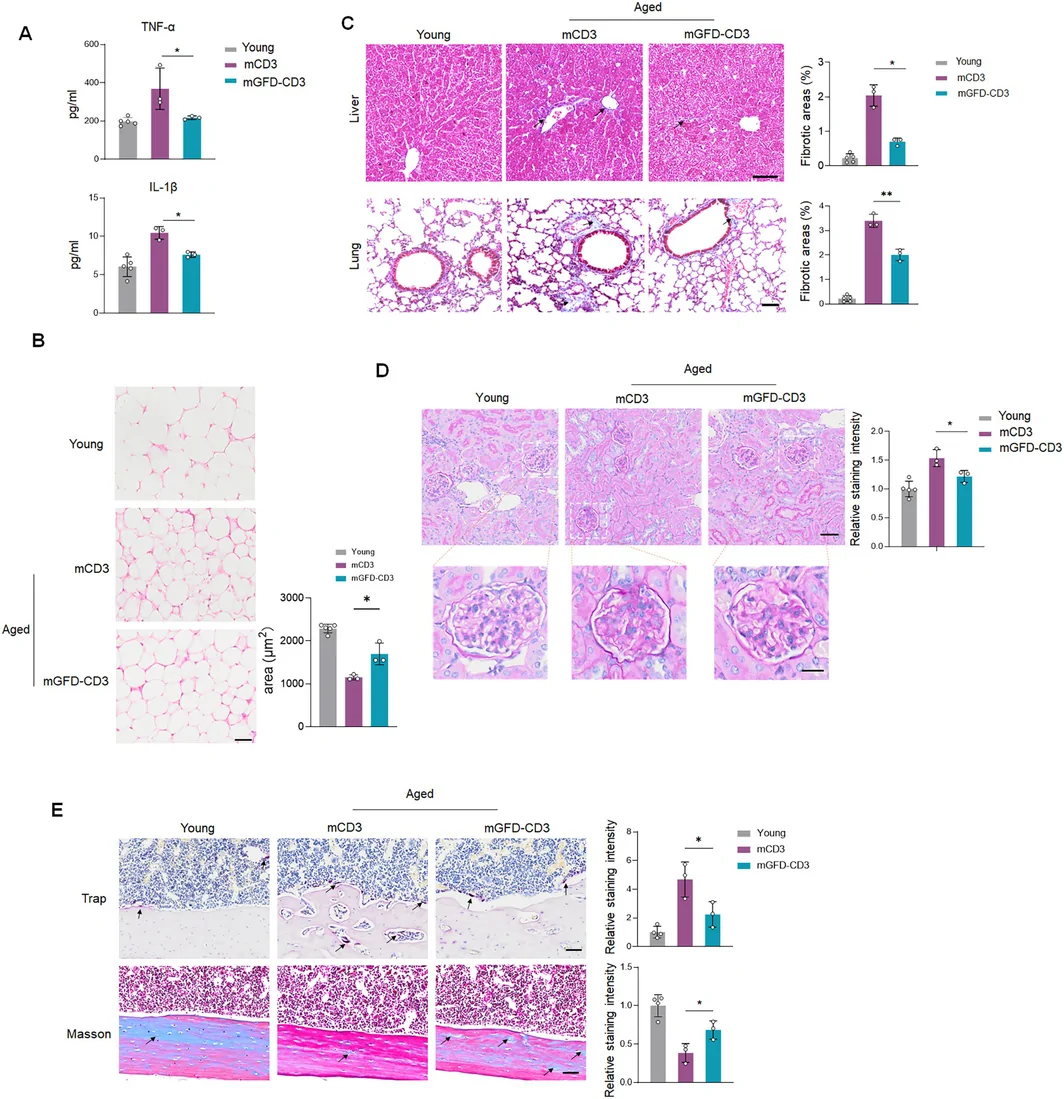
Elimination of senescent cells with mGFD‐CD3 ameliorated age‐related phenotypes in mice. (A) ELISA analysis for TNF‐α and IL‐1β in blood samples collected from mice treated with mGFD‐CD3 or mCD3 at 3 Months. (*n* = 5 for young group; *n* = 3 for mCD3 group; *n* = 3 for mGFD‐CD3 group). (B) (*left*) H&E staining of IAT samples collected from mice described in (a). Quantification of adipocyte areas (*right*). Each data point represents an individual mouse. Scale bar, 50 μm. (C) (*left*) Masson staining of liver and lung tissues collected from the mice described in (a). Black arrows indicate positive staining. Scale bar, 50 μm. (*right*) Quantification of the fibrotic areas of liver or lung tissues using Image J. (D) PAS staining of kidney samples from mice described in (a). Scale bar, 50 μm (original) and 15 μm (magnified). Quantification was achieved using Image J. (E) TRAP and Masson staining of femur samples from mice described in (a). Black arrows indicate positive staining. Scale bar, 50 μm. Quantification was achieved with Image J. Data are presented as mean ± SD and analyzed by one‐way ANOVA followed by Tukey's multiple comparisons test (A–E). *p ≤ 0.05.

mGFD‐CD3 treatment ameliorated age‐related pathologies in mice, prompting further investigation into their functional improvements. Treated mice exhibited notable improvements in various motor abilities, including strength, endurance, and running speed, after 6 months (Figure 5A). Consistently, we detected increases in skeletal muscle fiber density, which suggested mitigation of muscle loss (Figure 5B). Quantitative micro‐CT analysis of the trabecular bone of the distal femur in treated mice revealed a reduction in bone loss compared to control (Figure 5C). This effect may be due to mGFD‐CD3‐induced improvements in the bone microenvironment (Figure 4E).

**FIGURE 5:**
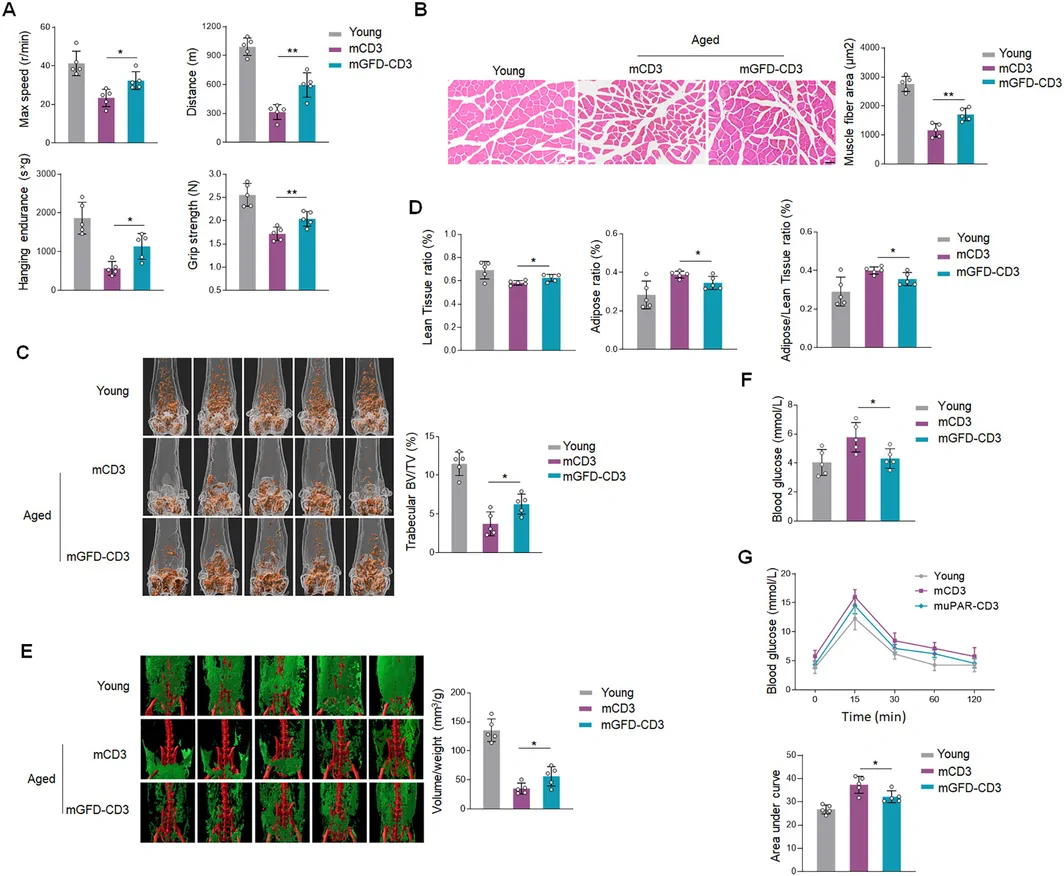
Physical attributes of naturally aged mice were enhanced following mGFD‐CD3 treatment. (A) Maximal walking speed, treadmill endurance, hanging endurance, and grip strength of naturally aged mice treated with mCD3 or mGFD‐CD3 were recorded at 6 months after the first infusion (*n* = 5 mice per condition). (B) H&E staining of gastrocnemius collected from mice treated with mCD3 or mGFD‐CD3, sampled 6 months after the first infusion. Scale bar, 50 μm. Relative cell areas in the gastrocnemius were quantified using Image J (*n* = 5 mice per condition). (C) Representative micro‐CT images of the distal femurs of the mice treated with mCD3 or mGFD‐CD3 at 6 months after the first infusion. The relative bone volume fraction (BV/TV) of cancellous bone in the distal femur was quantified for each sample and analyzed by Imaris image analysis software (*n* = 5 mice per condition). (D) The percentages of total lean mass and total adipose mass and the adipose mass/lean mass ratio of naturally aged mice treated with mCD3 or mGFD‐CD3 6 months after the first infusion were recorded using InAlyzer software (*n* = 5 mice per condition). (E) (left) Micro‐CT images of SAT and bone of the naturally aged mice treated with mCD3 or mGFD‐CD3 were recorded 6 months after the first infusion. Green, SAT; red, bone. (right) Quantification of the adipose tissue of mice based on the micro‐CT images (*left*). Data were normalized to mouse body weight (*n* = 5 mice per condition). (F) Basal glucose levels after 12 h of starvation in mice treated with mCD3 or mGFD‐CD3 at 6 months after the first infusion (*n* = 5 mice per condition). (G) (*upper*) Levels of glucose before (0 min) and after the intraperitoneal administration of glucose (2 g per kg body weight) (samples as per in (f)). (*lower*) Area under the curve (AUC) for the results from (g) (*n* = 5 mice per condition). The mouse experiments are representative results from two independent experiments. Data are presented as mean ± SD and analyzed by one‐way ANOVA followed by Tukey's multiple comparisons test (A–G). *p ≤ 0.05; **p ≤ 0.01.

Aging is associated with the ectopic distribution of adipose tissue, which is characterized by a reduction in subcutaneous adipose and an increase in visceral fat (Li et al. 2021; Nguyen and Corvera 2024). Therefore, we evaluated body fat percentage, the size of subcutaneous adipocytes, and the volume of abdominal subcutaneous adipose tissue via dual‐energy X‐ray and micro‐CT analysis. Compared to the control group, treatment with mGFD‐CD3 significantly increased the percentage of lean tissue in aged mice and reduced the ratio of adipose tissue to lean mass (Figure 5D). These findings are consistent with the observed increase in muscle fiber cross‐sectional area. Notably, although we found that the overall body fat percentage decreased in mice treated with mGFD‐CD3, the average adipocyte area and the volume of abdominal subcutaneous adipose tissue increased (Figure 5E, Figure S11F). These findings suggested that the clearance of senescent cells in mice mediated by mGFD‐CD3 promoted healthier fat distribution and, consequently, may have contributed to improved metabolism.

Given that senolytics have been shown to improve glucose tolerance in aged mice, we measured glucose metabolism in mGFD‐CD3‐treated aged mice. Following a 12‐h fasting period, we noted that blood glucose levels in the treatment group were lower than those in the control group (Figure 5F). Furthermore, the results of intraperitoneal glucose tolerance tests (IPGTT) indicated that blood glucose levels in the treatment group returned to baseline more rapidly than in controls after glucose challenge (Figure 5G). Correspondingly, aged mice treated with mGFD‐CD3 showed improved peripheral insulin sensitivity (Figure S11G), consistent with findings from previous antiaging studies using uPAR‐directed CAR T cells 2.5 months after treatment (Amor et al. 2024). These findings suggest that treatment with the engager significantly improved glucose‐insulin homeostasis in aged mice. Therefore, these results indicate that targeted elimination of senescent cells by mGFD‐CD3 leads to a significant improvement in age‐related phenotypes in mice.

### 2.5 The Safety of hGFD‐CD3 in Aged Nonhuman Primates

We investigated the potential for hGFD‐CD3 as a senolytic therapy in naturally aged rhesus macaques, a nonhuman primate with significant phylogenetic similarity to humans, including their physiological and anatomical traits. The amino acid sequences of uPAR are highly homologous between humans and rhesus macaques, and human uPA has been reported to have good cross‐reactivity with macaque uPAR (Engelholm and Behrendt 2001; Rhesus Macaque Genome et al. 2007). A binding assay revealed good binding of hGFD‐CD3 to rhesus macaque T cells (Figure 6A).

**FIGURE 6:**
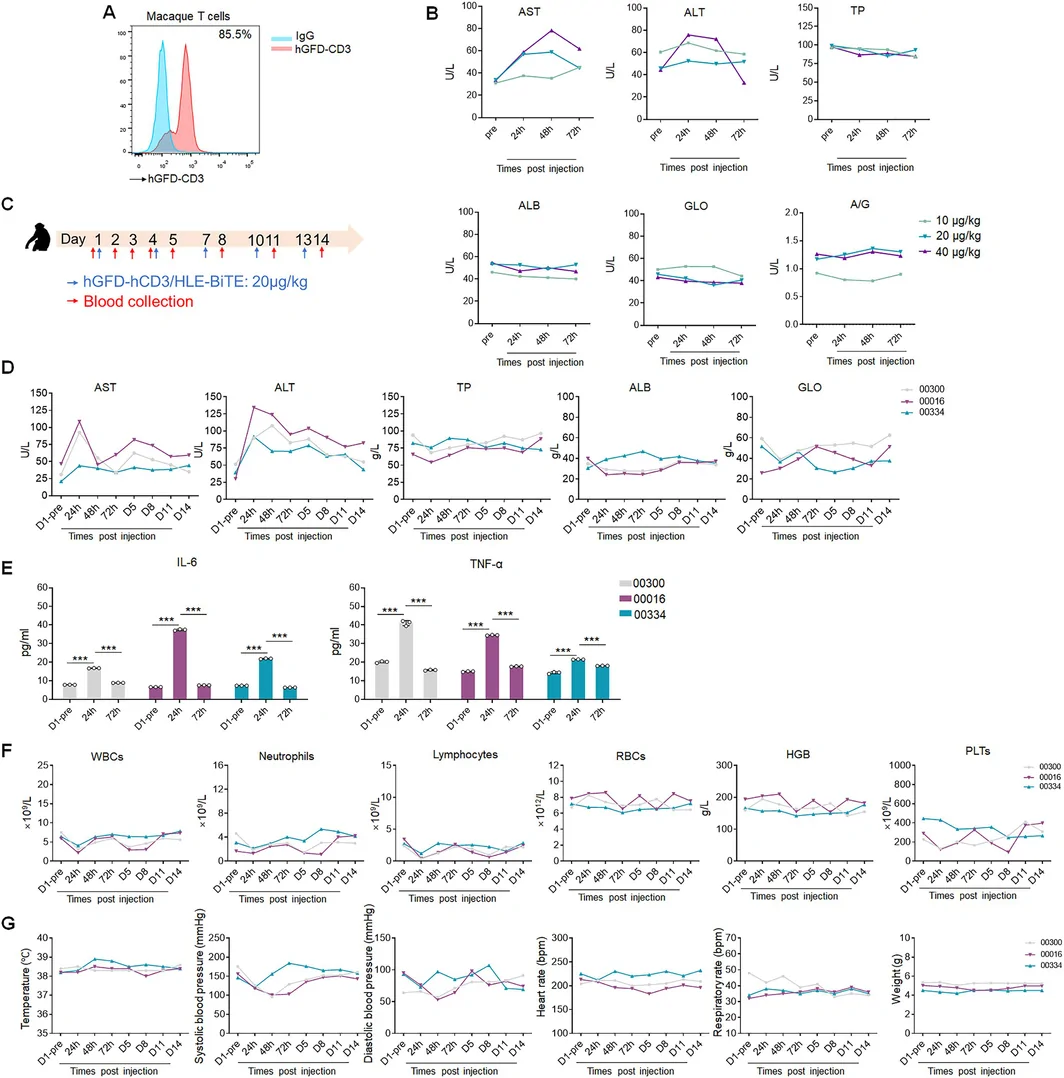
Assessing safety attributes of hGFD‐CD3 treatment in aged nonhuman primates. (A) Semiquantitative analysis of cell surface binding of hGFD‐CD3 to macaque T cells via flow cytometry. (B) Monitoring of liver function after administration of each different dose within a 72‐h period. (C) Schematic diagram of the experiment to investigate clearance of senescent cells from macaques, where hGFD‐CD3 was administered at a dose of 20 μg/kg every three days. (D) Liver function was evaluated throughout the injection phase. (E) Changes in cytokine levels in serum samples from three macaques before, as well as 24‐ and 72 h after the first injection of hGFD‐CD3, as determined via ELISA. Data are presented as mean ± SD of triplicate samples analyzed by two‐tailed unpaired Student's *t*‐test with Welch's correction. (F) Hematological parameters were assessed throughout the injection phase. (G) Six accessible vital sign parameters were continuously monitored during the injection period. Data is presented as mean ± SD of triplicates and analyzed by two‐tailed unpaired Student's t test with Welch's correction (E). ***p ≤ 0.001.

We began with a single‐dose escalation trial of hGFD‐CD3 dosages administered via intravenous injection in a 24‐year‐old macaque (00334), an age roughly equivalent to a 70‐year‐old in human (Yang et al. 2023). Given that a dose of 100 μg/kg in mice corresponds to an approximate dose range of 25–35 μg/kg in macaques (Nair et al. 2018), we therefore tested doses ranging from 10 μg/kg to 40 μg/kg in the macaque. No abnormalities were observed at doses of 10 and 20 μg/kg, with the exception of a slight increase in AST levels at 20 μg/kg (Figure 6B). At a dose of 40 μg/kg, the macaque showed a slightly impaired mental state, anorexia, and a 50%–100% increase in AST and ALT levels (Figure 6B), although these levels were still within the normal reference range (Qiao et al. 2009). Consequently, a dose of 20 μg/kg was selected for subsequent experiments.

Two 24‐year‐old macaques were injected with a dose of 20 μg/kg of hGFD‐CD3 (Figure 6C). Despite the presence of elevated ALT and AST levels within the normal range (Figure 6D) and an approximate doubling of IL‐6 and TNF‐α (Figure 6E), macaque 00300 exhibited no obvious clinical signs. However, macaque 00016 exhibited anorexia and a 100%–400% elevation in AST and ALT levels that marginally exceeded the normal reference range after an initial injection, with AST levels peaking at 108 U/L and ALT levels peaking at 134 U/L (Figure 6D). The level of the cytokine IL‐6 in this macaque was elevated by approximately 4‐fold, which was greater than the levels observed in the other two macaques (Figure 6E). These abnormalities cleared within 3 days (Figure 6E).

We assessed blood cell counts, hemoglobin levels, platelet counts and six accessible vital sign parameters: respiratory rate, heart rate, systolic blood pressure, diastolic blood pressure, body temperature, and weight on the second day following the 20 μg/kg injection. Decreases in white blood cell counts, neutrophil counts, lymphocyte counts, and blood pressure were observed in the macaques. However, by the third day post‐injection, these parameters returned to baseline levels (Figure 6F,G). This pattern is similar to the described mechanism of BiTEs in humans, where white blood cells migrate from circulation into tissues to engage target cells (Goebeler et al. 2016; Locatelli et al. 2021). In addition, the observed fluctuations in hematological parameters and vital signs decreased and then stabilized during subsequent treatments (Figure 6F,G). This finding indicates that repeated treatment does not present significant safety concerns, as uPAR‐positive cells are eliminated and adverse signs are merely transient.

### 2.6 hGFD‐CD3 Eliminates Senescent Cells in Nonhuman Primates

To explore the efficacy of the engager, we collected blood, skin, fat, and muscle tissue samples at different time points for up to 90 days. These tissues were selected due to their sensitivity to aging‐related changes and can be obtained through minimally invasive surgical procedures to reduce the lesions on the monkeys. There were no significant alterations in blood cell counts, hemoglobin levels, platelet counts, liver function parameters, or vital signs on Days 42 and 90 (Figure S12). Considering that adipose tissue is prone to aging over time, we used the subcutaneous adipose tissue (SAT) sections to label senescent cells via SA‐β‐gal staining. Treatment with hGFD‐CD3 decreased the number of senescent cells on Day 42; however, the numbers of senescent cells increased on Day 90 (Figure 7A). The expression of the senescence target uPAR and the senescence markers P16^INK4a^ and P21 on SAT, skeletal muscle (SKM), and skin decreased on Day 42 and recovered on Day 90, as detected by qPCR (Figure 7B,C). In findings reminiscent of observations from our mouse experiments, we detected reduced expression levels of markers associated with immune cells, including macrophages, T cells, and NK cells, which indicated a decrease in the abundance of these immune cells within the tissues (Figure 7D). Consistently, the concentration of serum SASPs significantly decreased following treatment with hGFD‐CD3 and returned to baseline levels by Day 90 (Figure 7E). These results suggest hGFD‐CD3 successfully cleared senescent cells and reduced chronic inflammation in macaques, with senescent cells re‐accumulating roughly 3 months post‐treatment. We also observed that the accumulation of senescent cells was highest in macaque 00016, potentially explaining the marked short‐term variations in transaminases and cytokines after hGFD‐CD3 treatment. In conclusion, our data suggest that hGFD‐CD3 functions as a senolytic agent to deplete senescent cells in vivo without severe side effects in aged nonhuman primates.

**FIGURE 7:**
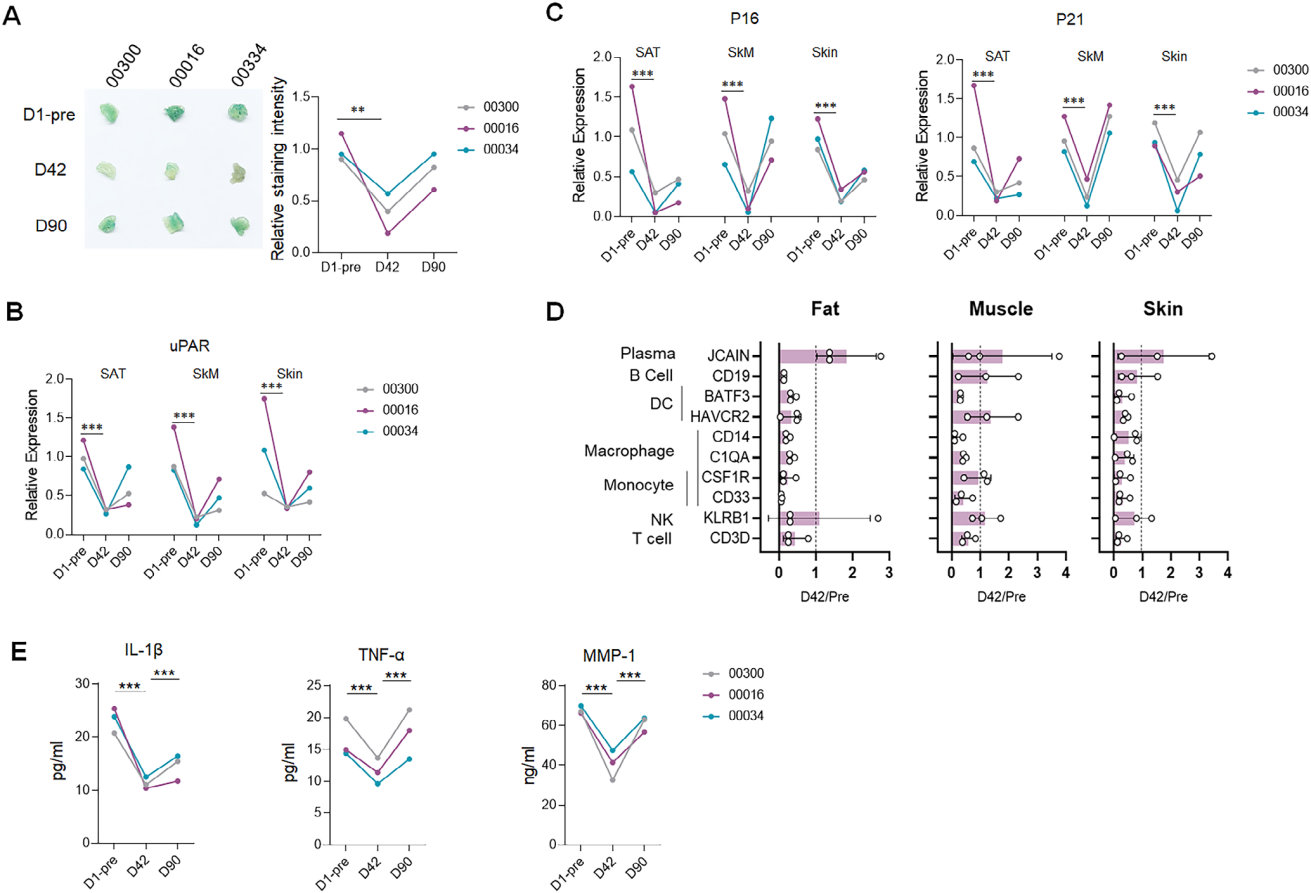
hGFD‐CD3 clears senescent cells in aged nonhuman primates. (A) SA‐β‐Gal staining of subcutaneous adipose tissue and quantification before injection, as well as at Days 42 and 90 after the first injection. (B) Fold‐changes in mRNA expression levels of uPAR in subcutaneous adipose tissue, muscle, and skin before injection and at Days 42 and 90 after the first injection. (C) Fold‐changes in mRNA expression levels of the senescence markers P16^INK4a^ and P21 in subcutaneous adipose tissue, muscle, and skin before injection and at Days 42 and 90 after the first injection. (D) The expression levels of representative genes from T cell‐, NK‐, Monocyte‐, Macrophage‐, DC‐, B cell‐ and Plasma‐cell types in liver, fat, and skin tissue collected from three macaques before injection, and at Days 42 and 90 after the first injection of hGFD‐CD3, and the data of pre‐administration were normalized and compared to those of the data of day 42. (*n* = 3). (E) Changes in cytokine levels in serum samples from three macaques before injection, and at Days 42 and 90 after the first injection of hGFD‐CD3, as determined via ELISA. Data is presented as mean ± SD of triplicates and analyzed by paired two‐tailed Student's *t*‐test. **p ≤ 0.01; ***p ≤ 0.001.

## 3 Discussion

uPAR has been recognized as a potential target for antiaging interventions. However, while uPAR‐targeted senolytic CAR‐T cells are effective, their activity against uPAR‐expressing healthy tissues can lead to dose‐dependent toxicities. Although these side effects may be mitigated by dose adjustment in laboratory mice, clinical translation to humans is complicated by inter‐patient heterogeneity. Factors, such as age, genetic background, and physiological status significantly influence CAR‐T cell expansion kinetics, which amplifies the safety risks in clinical application. In contrast, BiTE therapies offer superior dose‐titratability and a “hit‐and‐run” pharmacokinetic profile, due to the significantly shorter half‐life in vivo compared to CAR‐T cells. Given their transient mode of action, the safety profile of BiTE therapies is enhanced by their dosing flexibility—allowing for rapid discontinuation upon toxicity or a staggered schedule (e.g., during wound healing) to reduce side effects. Here, we constructed GFD‐CD3 BiTE to eliminate uPAR‐positive senescent cells and identified hepatotoxicity as the primary safety concern associated with uPAR‐targeted therapies. This toxicity stems from uPAR expression on liver endothelial cells and was effectively monitored through liver function markers such as serum ALT and AST. Following optimized dosing, GFD‐CD3 can effectively clear senescent cells in aged mice and non‐human primates, conferring broad benefits such as attenuated tissue fibrosis, reduced bone loss, improved metabolic function, and enhanced mobility. Based on our findings, we propose that GFD‐CD3 represents a viable senolytic therapeutic candidate, with predictable and monitorable hepatotoxicity that can be managed through dose titration and biomarker‐guided surveillance to ensure safe clinical application.

Previous studies have speculated that the side effects of high‐dose uPAR CAR T therapy may be linked to the local accumulation and activation of macrophages (Amor et al. 2020). However, we noted that NSG mice also exhibited hepatotoxicity after GFD‐CAR T cell therapy, whereas NSG mice typically do not show symptoms related to macrophage activation due to lack of functional macrophages (Giavridis et al. 2018; Manni et al. 2023). Our immunohistochemical analysis, together with published single‐cell sequencing data (Amor et al. 2024), demonstrates uPAR expression in hepatic endothelial cells. It is well established that damage to liver endothelial cells can induce microcirculatory disturbances, resulting in ischemia and hypoxia of hepatocytes, and subsequent liver injury (Hordeaux et al. 2024; Li et al. 2024; Zhang et al. 2022; Zhong et al. 2020). Therefore, we hypothesize that the side effects observed in our studies of injected mice resulted from the targeting of uPAR‐positive hepatic endothelial cells by uPAR‐directed BiTEs. This is supported by the conspicuous colocalization of mGFD‐CD3 with both uPAR and CD31 in the liver, along with a marked reduction in CD31^+^/uPAR^+^ cells after BiTE treatment. Consistent with this, the liver emerged as the primary site of toxicity following mGFD‐CD3 administration. Importantly, in uPAR‐inactivated mice, mGFD‐CD3 no longer induced hepatotoxicity or elevated serum transaminase levels. These observations suggest that liver function biomarkers may serve as sensitive indicators for monitoring the toxicity associated with the targeting of uPAR in antiaging therapies. Additionally, uPAR expression in liver endothelial cells increases with age (Amor et al. 2024), underscoring the critical need for rigorous hepatic monitoring in aged populations undergoing such therapies.

Although uPAR is expressed in endothelial cells of other tissues such as the lung and kidney, the proportion of uPAR^+^ endothelial cells in these organs is exceptionally low. Furthermore, microvessels in these tissues are predominantly composed of continuous endothelium anchored by an intact, continuous basement membrane consisting of collagen and laminin. Although uPAR‐targeted therapy eliminates this minor cell population, the intact basement membrane can effectively maintain the physical structure of the vessels. It has been reported that after mild endothelial injury, surrounding endothelial cells can migrate laterally and proliferate, promptly repairing endothelial defects, thereby causing no obvious organ functional impairment at the macroscopic level (Huang et al. 2016). In contrast, liver microvasculature is primarily composed of liver sinusoidal endothelial cells (LSECs), which possess unique fenestrae to ensure efficient material exchange and naturally lack a complete basement membrane. Given the high abundance of uPAR^+^ endothelial cells in the liver, GFD‐CD3–mediated clearance results in widespread rather than focal endothelial depletion. In the absence of basement membrane support, such extensive LSEC loss can readily trigger the collapse of the sinusoidal architecture and microcirculatory disturbances, which likely accounts for the selective hepatotoxicity induced by GFD‐CD3.

uPAR is also expressed in hematopoietic immune cells, including neutrophils and monocytes (Plesner et al. 1994). Therefore, we examined the numbers of these cells after dosing. Peripheral blood neutrophil levels showed a transient decrease at 6 h after dosing, whereas monocyte and eosinophil counts showed no significant changes throughout the monitoring period. This transient neutropenia induced by a non‐myeloid‐targeting BiTE has also been widely reported in other clinical bispecific antibody therapies (Ahn et al. 2023), and is generally attributed to peripheral redistribution of neutrophils caused by immune system activation rather than target‐mediated cell lysis. Consistent with this explanation, in our experiments, although peripheral blood neutrophils showed a significant decrease, there was no massive surge in systemic proinflammatory cytokines, and neutrophil counts rapidly returned to baseline within 24 h. However, because uPAR expression is upregulated with neutrophil differentiation and maturation, it cannot yet be completely ruled out that GFD‐CD3 exerts a cytotoxic effect on a small subset of mature neutrophils with high uPAR expression. The impact of neutrophil depletion on liver injury is dual: it can attenuate part of the liver injury in the early stage of acute liver injury, but may also impede liver repair in the later stage (Cartwright et al. 2025; Liu et al. 2006). Therefore, it remains to be further investigated whether GFD‐CD3 targets neutrophils and whether neutrophil depletion affects GFD‐CD3‐induced liver injury.

The effects of BiTE are dependent on endogenous T cells. Although T cell function declines with age, clinical evidence from older cancer patients receiving BiTE therapy indicates that endogenous T cells can be effectively engaged with BiTE and mount anti‐tumor responses (Aureli et al. 2025; Dorff et al. 2024; Lopez et al. 2025). It cannot be ruled out that immunosenescent older patients may require dose‐intensification strategies (e.g., increased dosage or frequency) to achieve therapeutic efficacy, a topic for further investigation. However, given that senescent cells in the body are relatively rare, non‐proliferative, and take months to reaccumulate after clearance, we speculate that doses lower than those used in clinical oncology may be sufficient for effective senolytic activity.

A recent report demonstrated that a synthetic uPA chimeric peptide, E16‐uPA_24_, could bind uPAR and glutamic acid receptors (Ming et al. 2024), thereby activating NK cells to attack senescent cells in vivo. Owing to its relatively low affinity to uPAR and glutamic acid receptors, the dosage of the chimeric peptide is relatively high. In contrast, the engager GFD‐CD3 binds endogenous T cells and senescent cells with a higher affinity, allowing effective clearance at a lower dosage. Furthermore, E16‐uPA_24_ requires prolonged and frequent dosing (e.g., three times weekly for 2 months in aged mice) to achieve senolytic effects, likely due to its small molecular size and rapid systemic clearance. By comparison, GFD‐CD3 effectively clears senescent cells with just once‐weekly dosing over 3 weeks. Given that previous studies have shown that periodic clearance of senescent cells (e.g., once every 6 months) is sufficient to alleviate aging‐related phenotypes, GFD‐CD3 therapy could, in theory, impose minimal treatment burden on patients.

In this study, we used a non‐orthotopic tumor model to evaluate the effects of GFD‐CAR T cells. Admittedly, this model can hardly reproduce the microenvironment and organ‐specific interactions unique to orthotopic tumors, and therefore has certain limitations in assessing the overall antitumor efficacy of GFD‐CAR T cells and their regulatory effects on the tumor microenvironment; if their antitumor effects are to be further investigated in depth in the future, it will still be necessary to introduce an orthotopic tumor model. However, the core question of this study was whether the hepatotoxicity induced by uPAR‐targeted immunotherapy is associated with macrophages. In this experimental system, the tumor‐bearing state mainly serves as a source of target antigen stimulation to drive the in vivo expansion and functional activation of GFD‐CAR T cells. Therefore, the specific anatomical location of the tumor has a relatively limited impact on answering the core mechanistic question of “whether macrophages are involved in the development of hepatotoxicity.”

To date, senolytic drugs that have entered clinical trials are small‐molecule compounds, including dasatinib combined with quercetin (D+Q), fisetin, ABT‐263, and UBX0101. Despite high initial expectations, their clinical efficacy has generally fallen short of predictions, often accompanied by toxic side effects such as diarrhea, anemia, and rash. This limitation is primarily attributed to the insufficient targeting specificity of most small‐molecule senolytics, which severely restricts their widespread application in antiaging therapy (Alum et al. 2025). uPAR is expressed on the surface of various types of senescent cells. Currently, uPAR‐targeted CAR‐T cells, CAR‐macrophages, and chimeric peptides have all demonstrated significant senolytic effects; furthermore, our research findings confirm that uPAR‐targeted BiTEs also possess potent anti‐aging activity, with the added advantage of dose‐titratability. These advances indicate that uPAR‐targeted immunotherapy may represent a key direction for achieving clinical breakthroughs in current senolytic drugs. Notably, uPAR is also expressed in some normal cells (Amor et al. 2024), making the safety of uPAR‐targeted therapies a primary concern. Our results demonstrate that senescent cells can be cleared in mice and non‐human primates while ensuring safety by monitoring liver function indicators. Nonetheless, the clinical translation of GFD‐CD3 warrants a cautious approach. For preventive applications aimed at extending healthspan, the risk–benefit ratio requires careful evaluation. However, for aging‐related diseases that already pose clear health threats, such as chronic kidney disease, Alzheimer's disease, and liver fibrosis, clinical need is more urgent. Therefore, prioritizing these diseases as indications for clinical exploration may be more reasonable.

## 4 Materials and Methods

### 4.1 Cell Culture

Human pancreatic cancer AsPC‐1 cell line (Kunming Cell Bank, Kunming Institute of Zoology, Chinese Academy of Science) and human breast cancer cell line MDA‐MB‐231 (Cell Bank of Type Culture Collection of the Chinese Academy of Sciences) were used in this study. Human embryonic kidney 293T (HEK‐293T) cells were sourced from our existing laboratory stocks. Human embryonic kidney Expi293F (HEK‐Expi293F) cells were purchased from Thermo Fisher Scientific (Thermo Fisher Scientific). The cell lines were tested via short tandem repeat (STR) profiling. Luciferase‐expressing cell lines were generated via infection with pTomo‐CMV‐luciferase‐IRESpuro lentivirus and subsequent selection with puromycin (Gibco, 1 μg/mL) for 2 weeks. Cultured HEK‐293T cells were transduced with either a plasmid vector designed for stable overexpression of human uPAR (G109913, NM 002659, youbio), mouse uPAR (EX‐Mm04529‐Lv205, NM 011113, iGeneBio), or an empty vector and then selected with puromycin to enrich the stably transfected cells. As the AsPC‐1 cell line exhibits high expression of uPAR, it was infected with the shRNA lentiviral vector (5′‐CCACTTCCTGAAATGCTGCAA‐3′) or a scrambled shRNA lentiviral vector. The HEL1 cell line was purchased from the China Infrastructure of Cell Line Resources. All cell lines except HEK‐Expi293F were cultured in Dulbecco's modified Eagle's medium (DMEM, Thermo Fisher Scientific) supplemented with 10% fetal bovine serum, penicillin (100 U/mL), and streptomycin (100 mg/mL) (Thermo Fisher Scientific) at 37°C with 5% carbon dioxide (CO_2_). HEK‐Expi293F cells were cultured in Celer‐S201 293 serum‐free medium (bioengine) in a 5% CO_2_ incubator at 37°C and 120 rpm.

### 4.2 Isolation and Culture of Human T Cells and Generation of CAR T Cells

Primary T cells were isolated from the peripheral blood of three healthy donors with the RosetteSep Human T‐Cell Enrichment Cocktail (Stemcell Technologies) according to the manufacturer's protocol. The purity of the isolated cells was evaluated by flow cytometry using phycoerythrin‐conjugated anti‐human CD3 (BioLegend, 300408). T cells were cultured in RPMI 1640 medium (Gibco) supplemented with 10% FBS, 100 U/mL penicillin, 100 mg/mL streptomycin sulfate, and 200 U/mL IL2 (PeproTech). T cells were stimulated by human CD3/CD28 beads (Thermo Fisher Scientific) at a ratio of 1:1 for 72 h. The coding sequences for GFD or CD19‐specific scFv (FMC63) were fused to a CAR backbone that includes a human CD8 hinge spacer and transmembrane domain, a 4‐1BB costimulatory domain, CD3ζ and mKate2. The complete sequence of the CAR expression molecule was subsequently cloned into the lentiviral vector pTomo and expressed under the control of a CMV promoter. For all constructs, lentiviral supernatant production was performed according to previously published reports (Guo et al. 2024; Wang et al. 2024). To generate CAR T cells, T cells were cultured for 72 h and infected with lentiviral particles in the presence of LentiBOOST (Sirion Biotech). CAR expression, measured as the relative mKate2 signal ratio, was subsequently assessed 72 h later by flow cytometry. The study was approved by the Biomedical Ethics Committee of West China Hospital of Sichuan University (approval no. 2022151). Our protocol was in accordance with the principles of the Declaration of Helsinki.

### 4.3 Cytotoxicity Assessment of CAR T Cells and Cytokine Release Assay

The cytotoxicity of the CAR T cells was tested via a luciferase assay system (Promega). Briefly, 2 × 10^3^ target cells per well were seeded in 96‐well plates with 100 μL of medium, together with an equal volume of effector cells or control medium added according to the stated E:T ratios. After 24 h of coculture, the supernatant was collected and used to determine the concentrations of IFN‐γ (Invitrogen, KHC4021) and TNF‐α (Proteintech, KE00154). The target cells were then lysed to test the cytotoxicity of CAR T cells according to the manufacturer's instructions (Promega).

### 4.4 Cytotoxicity Assays of T Cells Mediated by GFD‐CD3

T cells were added to the 96‐well plates containing senescent cells at an effector‐to‐target (E: T) ratio of 1:1 in the presence of varying concentrations of GFD‐CD3 BiTE or negative control. After 10 h of coculture, the culture supernatant was collected for measurement of cytokine concentrations. For cytotoxicity assays in adherent target cells, the suspended cells were removed by gentle aspiration, and the adherent cells were stained with Hoechst (Sigma‐Aldrich, 1 μg/mL) for 10 min after fixation and counted with an Opera Phenix Plus high‐content imaging system (Revvity).

### 4.5 In Vivo NSG Mouse Tumor Models

In all in vivo studies, 6‐ to 8‐week‐old female NOD/ShiLtJGpt‐Prkdc^em26Cd52^Il2rg^em26Cd22^/Gpt (NSG) mice (GemPharmatech Co. Ltd.) were used. A total of 5 × 10^5^ AsPC1‐luciferase cells were injected subcutaneously into NSG mice to establish xenograft models. Mice were subjected to intraperitoneal injection of 150 mg/kg d‐luciferin and potassium salt (BioVison) 10 min before they were placed in a chamber supplied with a 3% isoflurane gas mixture with oxygen (RWD) for 2 min. The tumor burden was then measured with the IVIS Spectrum (Lumina XR, PerkinElmer) and analyzed with the Living Image software (Caliper Life Science). Subsequently, 5 × 10^6^ CD19 CAR T cells or GFD‐CAR T cells, at the indicated doses, were administered intravenously via tail vein injection on Day 5 following tumor cell injection. All animal studies were approved by the animal ethics committees of the West China Hospital of Sichuan University (approval no. 20220517002).

### 4.6 Construction, Expression, and Purification of hGFD‐CD3 and mGFD‐CD3

hGFD‐CD3 and mGFD‐CD3 were constructed with recombinant DNA technology. The hGFD‐CD3 coding sequences for the variable heavy (VH) and variable light (VL) chain regions of the human CD3 antibody (hCD3), as well as the coding sequence of human GFD (hGFD), were derived from previously published sequences (Deegen et al. 2021; Kumar et al. 2022). Similarly, the coding sequence of mGFD‐CD3 was derived from the mouse CD3 antibody (mCD3) antibody and the mouse GFD (mGFD) (Lin et al. 2010; Nouveau et al. 2021). We incorporated a flexible (G_4_S)_3_ linker sequence between the GFD and anti‐CD3 scFv fragments. The molecules were fused to an immunoglobulin G (IgG) crystallizable fragment (Fc) domain to generate a half‐life extended (HLE) BiTE. In addition, the molecules were labeled with a 6× His‐tag at the carboxyl terminus. Additionally, we performed fusion of the aforementioned anti‐hCD3 or anti‐mCD3 scFv and Fc to construct the hCD3‐binding control protein and the mCD3‐binding control protein as negative controls. The sequences encoding these fragments were subcloned and inserted into the pCDH‐CMV‐MCS‐EF1a‐copGFP vector, which was subsequently transfected into HEK‐Expi293F cells. The supernatant of HEK‐Expi293F cells was collected, filtered through a 0.45‐μm filter and purified using the AKTA pure chromatography system (GE Healthcare). Purification of the monomeric protein was performed via a process involving immobilized nickel chelate chromatography. The quality of BiTE was assessed via Coomassie brilliant blue staining and Western blot analysis. A monoclonal anti‐6x His‐tag antibody (Cell Signaling Technology, 12698) and a secondary peroxidase‐conjugated goat‐anti‐rabbit IgG antibody (Sigma, A6154) were used for the Western blot analysis.

### 4.7 Bispecific Antibody Binding Assay

Binding studies of GFD‐CD3 and the negative control with senescent cells with high uPAR expression and CD3^+^ T cells were carried out by flow cytometry and immunofluorescence. For flow cytometry, briefly, cells were incubated with the engager and the anti‐6x His‐tag antibody (Cell Signaling Technology, 12698, 1:800 dilution). Next, the preparations were incubated with goat anti‐Rabbit IgG (H+L) Cross‐Adsorbed Secondary Antibody, Alexa Fluor 488 (Thermo Fisher Scientific, A11008, 1:1000 dilution), and the fluorescence of the stained cells was measured on a flow cytometer. For immunofluorescence, senescent cells were induced on coverslips in 24‐well plates. Cells were subsequently incubated with the engager and the anti‐6x His‐tag antibody for 1 h followed by washes and fixation with 4% PFA for 5 min. Thereafter, cells were washed three times and then incubated with goat anti‐Rabbit IgG (H+L) Cross‐Adsorbed Secondary Antibody for 1 h at room temperature. Nuclei were stained with DAPI (1 μg/mL, Sigma) for 10 min. Fluorescent images were captured using a confocal microscope (Nikon).

### 4.8 Flow Cytometry

The specified antigens were targeted using the following antibody clones: FITC‐conjugated anti‐human CD25 (clone BC96, BioLegend, 302604, 1:100 dilution), FITC‐conjugated anti‐human CD69 (clone FN50, BioLegend, 310904, 1:100 dilution), PE‐conjugated anti‐human CD3 (clone SP34‐2, BD Biosciences, 552127, 1:100dilution), FITC‐conjugated anti‐mouse CD25 (clone 3C7, BioLegend, 101907, 1:100 dilution), PE‐conjugated anti‐mouse CD3 (clone 17A2, BioLegend, 100206, 1:100 dilution), and the anti‐uPAR antibody (clone VIM5, Invitrogen, 17‐3879‐42, 1:1000 dilution). For surface staining, a total of 1 × 10^6^ target cells were harvested and resuspended in cold PBS containing 2% FBS, with antibodies specific for cell surface antigens for 1 h at 4°C in the dark. After the incubation period, cells were washed twice with a wash buffer and acquired on the flow cytometer immediately. The specificity of the anti‐uPAR antibodies was confirmed via the use of a species‐matched control IgG All flow cytometry data were collected using a BD LSRFortessa system (BD Biosciences) and analyzed with FlowJo software (version 10.0). Details of the antibodies are presented in Table S1. The gating strategy for flow cytometric analysis was shown in Figure S13.

### 4.9 Western Blotting

Harvested tissues were lysed in RIPA buffer, and protein concentrations were quantified using BCA protein assay kits (Beyotime). Total protein lysates from each sample were separated by 10% sodium dodecyl sulfate‐polyacrylamide gel electrophoresis and transferred to PVDF membranes (Millipore, USA). The membranes were blocked in Tris‐buffered saline with 5% nonfat milk and then incubated at 4°C overnight with anti‐uPAR (Proteintech, 10286‐1‐AP, 1:1000) and anti‐GAPDH (Abcam, ab9485, 1:1000) dies diluted in 5% bovine serum albumin (TBST). All horseradish peroxidase (HRP)‐conjugated secondary antibodies were purchased from Sigma‐Aldrich and used at a working dilution of 1:5000 in working solution (A6154). The blots were visualized with a chemiluminescent HRP substrate (Millipore).

### 4.10 Mice and In Vivo Procedures

All the mice were maintained under specific pathogen–free conditions at a constant temperature (20°C–25°C) and humidity (50%–60%). C57BL/6N mice were purchased from Jiangsu Wukong Biotechnology Co. Ltd. Mouse T cells were isolated from single‐cell suspensions of splenocytes by mechanical disaggregation using the EasySep Mouse T‐Cell Isolation Kit (Stemcell Technologies) and activated with mouse CD3/CD28 Dynabeads (Thermo Fisher Scientific). When the mice reached the age of 20–24 months, they were treated with mGFD‐CD3 through tail vein injection. Before and after treatment, peripheral blood counts were performed using a veterinary hematology analyzer (Drew Scientific), and blood biochemical analyses were performed using a Roche Cobas 8000 modular analyzer (Roche Diagnostics). The cytokine IL‐6 was measured with an ELISA kit (Proteintech, KE10007). For the PK study, female C57BL/6 mice (20 months of age) were given mGFD‐CD3 at 0.1 mg/kg via intravenous injection. Blood was collected from the mice at various timepoints after a single injection and analyzed for plasma. Concentrations of mGFD‐CD3 using a His Tag ELISA Detection Kit (Yuan Xin). Pharmacokinetic parameters using a non‐compartmental modeling approach were calculated with the PKSolver software. Twenty‐one days after treatment, representative numbers of mice from each treatment group were euthanized by cervical dislocation, and tissues were collected to detect changes in the expression of senescence markers and uPAR. For the aorta, the vessel was isolated and immediately placed in ice‐cold PBS; under a dissecting microscope, all macroscopically visible adventitial fat and surrounding connective tissue were carefully removed with fine forceps and scissors, ensuring that only the purified arterial wall was used in subsequent experiments. Six months after treatment, the remaining mice were assessed to measure changes in age‐related phenotypes and physical function. Cytokine and SASP analyses were carried out with ELISA kits (Meimian). All experiments with mice were approved by the Ethics Committee of the West China Hospital of Sichuan University West China Hospital (approval no. 20240109002).

Genetically modified uPAR^*−/−*^ mice bred on a C57BL/6N background were kindly provided by Prof. Wang Ben (Ming et al. 2024). The housing conditions for all mice were maintained as previously described. The two female mice and one male mouse (6–8 weeks of age) were given mGFD‐CD3 at 0.3 mg/kg via intravenous injection. Blood was collected from the mice at stated timepoints after the injection and subjected to blood biochemical analyses. Fourteen days after treatment, mice were euthanized by cervical dislocation, and tissues were collected to detect changes in the expression of CD31 and uPAR.

### 4.11 RNA Isolation and qPCR Analysis

Total RNA was isolated from cells or tissues using TRIzol reagent (Sigma‐Aldrich), and DNA was removed using the TURBO DNAfree Kit (Thermo Fisher Scientific). The concentration of total RNA was assessed by spectrophotometry (NanoDrop One, Thermo Fisher Scientific). The RNA was reverse‐transcribed with random primers using the RevertAid First‐Strand Complementary DNA Synthesis Kit (Thermo Fisher Scientific). QPCR was performed in triplicate using SYBR Select Master Mix (Applied Biosystems) on a QuantStudio 3 system (Applied Biosystems). All the experiments were performed in strict accordance with the manufacturers' instructions. Relative expression was calculated using the 2^−ΔΔCT^ method with normalization based on 18 s ribosomal RNA expression. The DNA sequences for the primers used in this study are listed in Table S2.

### 4.12 RNA Sequencing and Data Analysis

RNA quality was tested using the Qseq400electrophoresis system, and samples with an RNA integrity number (RIN) score of greater than 7 were sent to the DNA Stories Bioinformatics Center. Preparation of mRNA libraries and mRNA sequencing was performed by the DNA Stories Bioinformatics Center using the MGI T7 instrument. The RNA‐seq data were aligned to the mice reference genome using software hista2 and subsequently analyzed for senescence signatures using the SenMayo module. The raw RNA‐seq data have been deposited in China National Center for Bioinformation (CNCB, https://www.cncb.ac.cn/services, subSAM150296).

### 4.13 Micro‐CT Imaging

Micro‐CT imaging of mice treated with mGFD‐CD3 or mCD3 was carried out with a micro‐CT imaging system (SkyScan 1276 System, Bruker). Before analysis, the mice were subjected to continuous anesthesia with 3% isoflurane/oxygen and were placed in a natural prone position on the micro‐CT bed, with anesthesia maintained using a 1.5% isoflurane/oxygen mixture. The scanning parameters for abdominal adipose tissue were 60 kV, 200 μA, and a 0.5 mm Al filter. The scanning parameters for the femur were 70 kV, 200 μA, and a 1.0 mm Al filter. All the analyses were performed via the manufacturer's evaluation software. The images were reconstructed with NRecon (version 1.7.4.2). 3D image analysis was performed with Imaris image analysis software (version 10.1.0).

### 4.14 Physical Function Assessment

Maximal walking speed was assessed with an accelerating rotarod system (Beijing Zhongshi Dichuang Technology Development Co. Ltd., ZS‐RDM‐XS). The apparatus was configured with a distance of 21 cm from the rod to the bottom plate, where a soft foam cushion was positioned to mitigate impact and prevent injury to the aged mice during falls. The mice were placed in separate lanes on a rod rotating at an initial speed of 4 rpm/min. The apparatus was set to accelerate from 4 to 60 rpm/min in 480 s. Briefly, the mice were allowed to acclimate to the rotarod system for three consecutive days. The maximal walking speed of the mice was tested for three consecutive days, beginning on the fourth day. The speed and time were recorded when the mouse fell from the balance bar. A treadmill test was performed on a motor‐driven treadmill (Beijing Zhongshi Dichuang Technology Development Co. Ltd., ZS‐PT‐III) to evaluate the endurance capacity. The treadmill was set at a 5° incline, and the mice were encouraged to run using 0.2 mA electrical stimulation, a parameter widely adopted in treadmill exhaustion tests to induce mild nociception without causing tissue damage (Marcaletti et al. 2011). The mice were allowed to acclimate to the treadmill for three consecutive days at 15 m/min for 5 min. After the mice were allowed to rest for 1 day, they ran on the treadmill at an initial speed of 5 m/min. The speed was increased from 5 to 15 m/min and then maintained until the mice were unable to keep running. The running time and running distance were recorded. The grip strength of all mouse limbs was measured using a grip strength meter (Beijing Zhongshi Dichuang Technology Development Co. Ltd., ZS‐ZL), set to peak mode. The experiment was repeated five times. The mice grasped a 0.5 mm wire with their forelimbs only, with the wire positioned at a height of 0.25 m above the surface of a biological safety cabinet. To prevent potential injury upon falling, a 1 cm‐thick soft cushion was placed on the benchtop. The time until the mice fell from the wire to the surface was recorded. Prior to the experiment, the mice were trained to grasp the wire for 3 days, with two sessions per day. Following a day of rest, measurements were taken once daily for five consecutive days, and the data were recorded.

### 4.15 Body Composition Measurements by Dual‐Energy X‐Ray Absorptiometry

Body composition was assessed with an InAlyzer DXA system (Medikors Inc.). Before analysis, mice were subjected to continuous anesthesia with 3% isoflurane/oxygen and were placed in a natural prone position on the InAlyzer scanning area, with anesthesia maintained via a 1.5% isoflurane/oxygen mixture. Each mouse was placed in the center of the InAlyzer scanning area in the prone position with the arms and legs extended out to the side and the tail straight out behind the body. The animal's head was positioned into a nose cone taped to the instrument's scanning area. The outcomes obtained using the InAlyzer software included total adipose mass (g), percentage of total adipose mass (%), total lean mass (g), and percentage of total lean mass (%).

### 4.16 Intraperitoneal Glucose Tolerance Test (IPGTT)

Blood samples from mice that had fasted for 12 h were collected at 0, 15, 30, 60, and 120 min after an intraperitoneal injection of glucose (2 g/kg body weight). The glucose concentrations were determined using a Yuwell 660 Blood Glucose Tester (Jiangsu Yuyue Medical Equipment & Supply Co. Ltd.).

### 4.17 Insulin Sensitivity and Glucose Tolerance Test (ITT)

Insulin (0.5 U/Kg) was injected intraperitoneally into mice after 4 h of fasting to test the insulin sensitivity (IST). Then, the blood glucose was measured at 0, 15, 30, 60, and 120 min. The glucose concentrations were determined using a Yuwell 660 Blood Glucose Tester (Yuyue).

### 4.18 Open Field Test

In brief, a mouse was positioned at the center of an empty apparatus measuring 50 cm × 50 cm with walls and an open top and permitted to explore the open field for a duration of 15 min. During this period, the mouse's spontaneous behavior was continuously recorded using an overhead CCD camera, and the total distance traveled was quantified through specialized software.

### 4.19 Bone Histomorphology and Tartrate‐Resistant Acidic Phosphatase (TRAP) Staining

Mouse femur bones were isolated and fixed in 10% neutral buffered formalin for 24 h. Bones were decalcified, embedded in paraffin, sectioned, and standard H&E and TRAP staining techniques were used for all bone sections at the histopathology research platform of Elite Hui Research Service. Images were collected using a Nikon ECLIPSE Ni‐E microscope (Nikon). To quantitate the TRAP‐positive cells and determine how much of their surface area covered the bone, we used the BioQuant software to quantitate osteoclasts per bone surface (Oc.S/BS and N.Oc/BS). We carried out these measurements by defining a region of interest with the stamp tool window. We then chose a diagonal line type and set the distance to the growth plate, and measured the Oc.S/BS and N.Oc/BS features for each section analyzed.

### 4.20 Histological Investigation

The tissues were fixed in a 4% paraformaldehyde solution, dehydrated using an ethanol gradient, cleared with xylene, embedded in paraffin, and sectioned into 4‐μm‐thick sections. These sections were then stained with hematoxylin and eosin (H&E) using a H&E Staining Kit (Beyotime). To assess the GBM, periodic acid‐Schiff (PAS) was used to estimate the glomerular deposition of matrix glycoprotein at the histopathology research platform of Elite Hui Research Service. To detect collagen fiber deposition, 4‐μm sections were stained with a Masson's trichrome staining kit (Beyotime) according to the manufacturer's instructions. After staining, the sections were examined and recorded under a Nikon ECLIPSE Ni‐E microscope (Nikon). Cell area measurements were quantitatively evaluated using ImageJ software (version 1.52a).

### 4.21 Senescent Cell Models

For etoposide‐induced senescence, cultured human lung fibroblasts HEL1 at approximately 60% confluence were exposed to 50 μM etoposide (MCE) for 36 h. Dimethyl sulfoxide (DMSO) was used as a vehicle control. For p16^INK4a^ overexpression–induced senescence, HEL1 cells at approximately 30% confluence were infected with a lentivirus produced from pTomo‐Teton‐P16^INK4a^‐T2A‐EGFP at an MOI of 50 in the presence of polybrene (8 μg/mL, Sigma‐Aldrich) to obtain HEL1‐P16^INK4a^ cells, which were then induced with doxycycline (2 μg/mL, Sigma‐Aldrich). DMSO, the vehicle of doxycycline, was used as a control. All senescent cell models were verified by SA‐β‐gal staining and then used for experiments.

### 4.22 Immunofluorescence Staining

Liver and lung samples were fixed in 4% buffered paraformaldehyde. Histological analyses were performed with standard protocols using paraffin‐embedded sections. The sections were then stained for immunofluorescence by blocking with 10% normal goat serum and 0.1% Tween in PBS for at least 1 h at room temperature. Then the sections were incubated with primary antibody at 4°C overnight. All primary antibodies used in this work were commercially available: uPAR (Proteintech, 10286‐1‐AP, 1:200), CD31 (Proteintech, 28083‐1‐AP, 1:400), F4/80 (CST, 70076S, 1:200), and Fc (Invitrogen, A18818, 1:200). The next day, the sections were washed with PBS three times and incubated with secondary antibodies and the tyramide signal amplification, then stained with DAPI. Fluorescent images were captured using a Vectra Polaris Automated Quantitative Pathology Imaging System. Stained areas were quantified using ImageJ software and an immunohistochemical profiler according to published methods.

### 4.23 Immunohistochemical Staining

Cells were induced in 24‐well plates, and upon completion of the induction process, the cells were fixed with 4% paraformaldehyde at 37°C for 15 min. The cells were washed twice with PBS and incubated with anti‐uPAR (Proteintech, 10286‐1‐AP, 1:200 in 2% FBS PBS) for 60 min at 37°C. The cells were subsequently washed twice with PBS and incubated in blocking buffer (2.5% BSA/10% goat serum/0.1% Tween‐20) for 1 h at 37°C. The samples were incubated with a goat anti‐Rabbit IgG (H+L) Cross‐Adsorbed Secondary Antibody, Cyanine3 (Thermo Fisher Scientific, A10520, 1:1000 dilution) in blocking buffer for 1 h at 37°C. After washing once with PBS, the cell nuclei were stained with DAPI (Sigma Aldrich) in PBS for 10 min in the dark. The cells were washed three times with PBS, mounted with Aqua‐Polymount on slides (Polysciences Inc., 18606‐20), and examined using a Nikon N‐STORM microscope (Nikon).

### 4.24 SA‐β‐Gal Staining

For senescent cells, adipose tissue and frozen tissue sections, SA‐β‐Gal activity was evaluated with a senescence β‐galactosidase staining kit (Cell Signaling Technology). The average staining intensity values of whole‐mount SA‐β‐gal staining of adipose tissues were calculated with ImageJ software (version 1.52a). All procedures were carried out according to the manufacturer's instructions.

### 4.25 Cell Immunofluorescence

Senescent and control cells were seeded in 24‐well flat‐bottom culture plates at a density of 2 × 10^4^ cells per well before fixation with 4% paraformaldehyde at 37°C for 15 min. The cells were washed twice with PBS and incubated with anti‐uPAR (Proteintech, 10286‐1‐AP, 1:200 in 2% FBS PBS) for 60 min at 37°C. The cells were subsequently washed twice with PBS and incubated in blocking buffer (2.5% BSA/10% goat serum/0.1% Tween‐20) for 1 h at 37°C. The samples were incubated with a goat anti‐Rabbit IgG (H+L) Cross‐Adsorbed Secondary Antibody, Cyanine3 (Thermo Fisher Scientific, A10520, 1:1000 dilution) in blocking buffer for 1 h at 37°C. After washing once with PBS, the cell nuclei were stained with DAPI (Sigma Aldrich) in PBS for 10 min in the dark. The cells were washed three times with PBS, mounted with Aqua‐Polymount on slides (Polysciences Inc., 18606‐20), and examined under a Nikon N‐STORM microscope (Nikon).

### 4.26 Cytotoxicity Assays

Senescent and control cells were seeded in 96‐well flat‐bottom culture plates in triplicate at a density of 5000 cells per well in RPMI 1640 with 10% fetal bovine serum, 2 mM glutamine, penicillin (100 U/mL), and streptomycin (100 mg/mL). After 24 h, the effector cells were added to each well at an effector–target (E:T) ratio of 1:1 in the presence of various concentrations of hGFD‐CD3 or the negative control. After 10 h of coculture, the culture supernatant was collected for measurement of cytokine concentrations. For cytotoxicity assays in adherent target cells, the suspended cells were removed by gentle aspiration, and the adherent cells were stained with Hoechst (Sigma‐Aldrich, 1 μg/mL) for 10 min after fixation and counted with an Opera Phenix Plus high‐content imaging system (Revvity).

### 4.27 Enzyme‐Linked Immunosorbent Assay (ELISA) for Cytokine Secretion

To examine the cytokine concentrations in the culture supernatants, the secretion of tumor necrosis factor α (TNF‐α) (Proteintech, KE00367) and interferon‐γ (IFN‐γ) (Invitrogen, KHC4021) were measured using ELISA kits specifically designed for these cytokines.

### 4.28 High‐Content Imaging and Video Analysis

High‐content imaging was performed every hour for a total of 10 h using an Opera Phenix Plus (Revvity) system with a 10× air immersion lens. Images collected each hour were stitched together to form a video. Nine fields of view were imaged for each 96‐well plate, with 6 z‐stacks per field at 2‐μm intervals, and the 6 z‐stacks were subjected to maximum projection to ensure comprehensive imaging of the cells. Image analysis was performed using Harmony (version 5.2).

### 4.29 Macaques and In Vivo Procedures

The three rhesus macaques used in this study were obtained from the Kunming Primate Research Center at the Kunming Institute of Zoology, Chinese Academy of Sciences and housed at the Association for Assessment and Accreditation of Laboratory Animal Care (AAALAC)‐accredited facility of the Primate Research Center of the Kunming Institute of Zoology. The rhesus macaque identification numbers were 00334 (female, 24 years old), 00016 (female, 24 years old), and 00300 (female, 24 years old). Macaque T cells were isolated from the peripheral blood using the non‐human primate T Cell Activation/Expansion Kit (Miltenyi Biotec). The macaques were treated with hGFD‐CD3 via intravenous injection. Before and after treatment, peripheral blood counts were performed using a veterinary hematology analyzer (Hemavet 950, Drew Scientific Inc.), and blood biochemical analyses were performed using the Dimension EXL 200 Integrated Chemistry System (Siemens). Cytokine and SASP analyses were carried out with ELISA kits (Shanghai Zhen Ke Biological Technology Co. Ltd.). SAT, SkM and skin samples were collected surgically before and 3 months after treatment. All surgeries were performed under aseptic conditions with the macaques under general anesthesia with a combination of ketamine (10 mg/kg, intramuscularly (i.m.)), sodium pentobarbital (20 mg/kg, i.m.), and atropine (0.05 mg/kg, i.m.). All protocols involving macaques were approved by the animal ethics committee of the Kunming Institute of Zoology, Chinese Academy of Sciences (approval no. ACUC‐PE‐2024‐01‐005). The experiments were performed in accordance with approved guidelines.

### 4.30 Statistical Analysis

All the statistical analyses were performed using GraphPad Prism statistical software (version 8.3.0). The data are presented as the mean ± SD. The statistical significance of differences between two groups was analyzed via unpaired Student's *t*‐test with the Welch correction. The statistical significance of differences among three or more groups was analyzed by one‐way ANOVA. Statistical significance was defined as *p ≤ 0.05, ** *p* ≤ 0.01, and ***p ≤ 0.001.

## Author Contributions

X. Zhao and D.Y. designed the study and was responsible for study supervision. J.D. and S.H. were responsible for the CAR T cell‐related experimental section. X. Zeng and S.Z. designed and prepared the BiTE proteins. X. Zeng and N.L. generated the human senescent cell models. X. Zeng and J.G. analyzed the cytotoxicity of GFD‐CD3 on senescent cells in vitro. X. Zeng, S.Z., D.Y., L. Li. and L. Lv detected the safety and effects of GFD‐CD3 in macaques. B.W. provided the uPAR knockout mice. X. Zeng, J.G., Y.Z. and N.L. detected the effects of GFD‐CD3 in aged mice. X. Zeng, X. Zhao, L.Y., B.W., and D.Y. analyzed the results and wrote the manuscript. All the authors have read and approved the published version of the manuscript.

## Funding

This work was supported by grants from Sichuan Science and Technology Program (2025NSFJQ0057 to D. Yang, 2026NSFSC0124 to X. Zhao), and Yunnan Science and Technology Talent and Platform Program (202405AD350052 to L. Li).

## Conflicts of Interest

The authors declare no conflicts of interest.

## Supporting information


**Figure S1:** Purification of recombinant protein from HEK‐293F cells.
**Figure S2:** hGFD‐CD3 could specifically bind to uPAR+ senescent cells and CD3+ human T cells.
**Figure S3:** Validation of T cell‐mediated cytotoxicity of HEL1 cells.
**Figure S4:** Establishing safe dosages of mGFD‐CD3 in naturally aged mice.
**Figure S5:** Distribution of mGFD‐CD3 and mCD3 in vivo.
**Figure S6:** Serum AST and ALT levels and tissue immunostaining studies in treated mice.
**Figure S7:** The damaging effects of mGFD‐CD3 treatment were abolished in uPAR knockout mice.
**Figure S8:** GFD‐CAR T cells eliminate uPAR‐positive cells in vitro and in vivo.
**Figure S9:** mGFD‐CD3 treatment alleviates systemic inflammation in aged mice.
**Figure S10:** Behavioraland physiological characteristics of mGFD‐CD3 treated mice.
**Figure S11:** Treatment with mGFD‐CD3 ameliorates age‐associated phenotypes in naturally aged mice 3 months after the initial injection.
**Figure S12:** Liver function, hematological parameters and six accessible vital sign parameters are continuously monitored on Days 14, 42, and 90, after the first injection.
**Figure S13:** Gating strategies.

**Table S1:** List of antibodies used in this study.

**Table S2:** Sequences of primers used in qPCR.

**Video S1:** It documents the process by which senescent cells are killed following the addition of 100 nM control BiTE to a co‐culture system of T cells and HEL1 senescent cells induced by p16 overexpression.

**Video S2:** It documents the process by which senescent cells are killed following the addition of 100 nM GFD‐CD3 BiTE to a co‐culture system of T cells and HEL1 senescent cells induced by p16 overexpression.

## Data Availability

The data that support the findings of this study are available on request from the corresponding author. The data are not publicly available due to privacy or ethical restrictions.
