# Supplementary material for: uPAR‐Targeting T Cell Engager Exerts Senolytic Effects in Mice and Non‐Human Primates With Serum Aminotransferase Activity as a Safety Monitor
Source: Aging Cell. 2026 Sep 28;25(10):e70742. doi: 10.1111/acel.70742 (PMC13620297; doi:10.1111/acel.70742)
Supplement: Supplementary file 1 [file ACEL-25-e70742-s001.docx]

**Supplementary Figures**

**Supplementary Figure 1. Purification of recombinant protein from HEK-293F cells.**

**A,** Coomassie blue staining of SDS/PAGE gel of the eluted CD3 protein (left) and Western immunoblot analysis of CD3 using an anti-His-tag antibody (right). **B,** Coomassie blue staining of SDS/PAGE gel of the eluted mGFD-CD3 protein (left) and Western immunoblot analysis of mGFD-CD3 using an anti-His-tag antibody (right). **C,** Coomassie blue staining of SDS/PAGE gel of the eluted hGFD-CD3 protein (left) and Western immunoblot analysis of hGFD-CD3 using an anti-His-tag antibody (right). M. Protein molecular weight markers. Data are representative of two independent experiments.

**Supplementary Figure 2. hGFD-CD3 could specifically bind to uPAR+ senescent cells and CD3+ human T cells.**

**A,** Representative images of β-galactosidase (SA-β-Gal) staining of different types of senescent cell models used in this study are shown. The quantification of SA-β-Gal-positive cells in five randomly chosen fields is shown on the right. Data is presented as mean ± SD and analyzed by one-way ANOVA followed by Turkey’s multiple comparisons test (****p* ≤ 0.001; ns, not significant). Scale bar, 200 μm. **B,** Immunofluorescence detection of uPAR on young or senescent cells by confocal fluorescence microscopy. Scale bar, 25 μm. **C,** Immunofluorescence studies showing cell surface binding of hGFD-CD3 to uPAR^+^ senescent cells. **D,** Immunofluorescence studies showing cell surface binding of hGFD-CD3 to human CD3^+^ T cells. Scale bar, 25 μm.

**Supplementary Figure 3. Validation of T cell-mediated cytotoxicity of HEL1 cells.**

Cytotoxicity was measured after co-culturing primary human T cells and untreated HEL1 cells. (**A**) or DNA damage HEL1 cells (**B**) at an E:T ratio of 1:1. Data are representative of two independent experiments. Data presented as mean ± SD and analyzed by unpaired two-tailed Student’s t-test with Welch’s correction. (***P ≤ 0.001; ns, no significant).

**Supplementary Figure 4. Establishing safe dosages of mGFD-CD3 in naturally aged mice.**

**A,** A schematic diagram of mGFD-CD3and a negative control (mCD3) used in this study. **B**, Representative images of β-galactosidase (SA-β-Gal) staining of different types of senescent cell models used in this study are shown. The quantification of SA-β-Gal-positive cells in three randomly chosen fields is shown on the right. Data is presented as mean ± SD and analyzed by one-way ANOVA followed by Turkey’s multiple comparisons test (***p ≤ 0.001; ns, not significant). Scale bar, 200 μm. **C,** Semiquantitative analysis of cell surface binding by mGFD-CD3 to young uPAR- MEF cells and uPAR+ MEF senescent cells via flow cytometry. **D,** Semiquantitative analysis of the cell surface binding of mGFD-CD3 to mouse CD3^+^ T cells via flow cytometry. **E,** Dose-response study of mGFD-CD3 and control mediated T cell cytotoxicity against senescent MEF cells (E: T=1: 1, 24 hours). **F,** Twenty-four hours after injection, the levels of AST and ALT in mouse serum were determined with a Cobas 8000 modular analyzer (*n*=2). **G,** At 24 hours after injection, ELISA was used to detect the release of the cytokine IL-6 in the serum of the mice (*n*=2). Each dot represents an individual mouse. **H**, Histopathological analysis of heart, lung, kidney and brain collected from the mice treated by mCD3 or mGFD-CD3. The data are presented as mean ± SD and analyzed by one-way ANOVA followed by Turkey’s multiple comparisons test (**E-G**) (***p* ≤ 0.01; ****p* ≤ 0.001; ns, not significant).

**Supplementary Figure 5 Distribution of mGFD-CD3 and mCD3 *in vivo*.**

**A,** The distribution of mGFD-CD3 in the major organs of naturally aged mice was quantitatively analyzed at 24h post-injection using ELISA (n = 2 for mCD3 group; n = 3 for mGFD-CD3 group). **B,** Representative images of immunofluorescence analyses performed in samples at 4h post-injection of BiTE to examine the colocalization of BiTE, CD31, and uPAR in lung tissues of naturally aged mice. Scale bar, 30 μm (original) and 15 μm (magnified).

**Supplementary Figure 6. Serum AST and ALT levels and tissue immunostaining studies in treated mice.**

**A,** The levels of AST and ALT in young mouse serum were determined with a Biochemical Analyzer before and after BiTE injection (n=3). **B,** Representative images of immunofluorescence analysis were performed at pre and 24h post-injection of BiTE to examine the colocalization of BiTE, CD31, and uPAR in the lung tissues of young mice (n = 3 for mCD3 group; n = 3 for mGFD-CD3 group). Scale bar, 30 μm (original) and 15 μm (magnified).

**Supplementary Figure 7. The damaging effects of mGFD-CD3 treatment were abolished in uPAR knockout mice.**

**A,** Immunofluorescence analysis was performed to examine the colocalization of CD31 and uPAR in the lung tissues of wild type mice (WT) and uPAR knockout mice (n = 3). Scale bar, 30μm. **B, C,** Histopathological analysis of liver, heart, lung, kidney and brain collected from the WT and uPAR knockout (*n* = 3). Scale bar, 50 μm. **D**, Serum AST and ALT levels in male mice 24 h after GFD-CD3 administration (n = 4). **E**, Representative H&E-stained images of the heart, liver, lung, kidney, and brain from male mice 24 h post-injection (n = 4), lesion areas are indicated by black arrows.

**Supplementary Figure 8. GFD-CAR T cells eliminate uPAR-positive cells *in vitro and in vivo*.**

**A,** Schematic presentation of the CAR domains (CD19-CAR as a control group). **B,** Representative CAR expression in T cells, detected by flow cytometric on day 3 after transduction. Percentages of positive cells are presented in the histograms. Data is representative of two independent experiments. **C,** Cytotoxicity was measured after coculturing primary human T cells transfected with the indicated lentivirus and target cells expressing luciferase at an effector-to-target (E:T) ratio of 5:1 for 24 hours, respectively. **D,** Nontransduced (NTD) cells, CD19 CAR T cells and GFD-CAR T cells were cocultured with senescence model of P16INK4a overexpression and DNA damage in erythroleukemic HEL-1 cells at a serial E:T ratio 24 hours. **E,** Cytokines in the coculture supernatant were quantified by ELISA after coculture at an effector-to-target (E: T) ratio of 1:1. **F,** QPCR was used to detect the expression of uPAR in HEK-293T-vector and HEK-293T-muPAR cells. **G,** Nontransduced cells (NTD), CD19 CAR-T cells and GFD-CAR-T cells were co-cultured with HEK-293T-vector and HEK-293T-muPAR cells at an E:T ratio of 5:1 for 24 hours. Cytotoxicity was assessed using a luciferase cytotoxicity assay and **H,** cytokines were measured by ELISA. **I,** (*Left*) NSG mice bearing AsPC1-luciferase xenografts were treated with 100 μL of saline, 5×10^6^ CD19-CAR T cells, or 5×10^6^ GFD-CAR T cells via tail vein injection. All mice were imaged with an IVIS imager at the indicated times. Tumor growth was assessed on the basis of total bioluminescence signals (*n* = 4). (*Right*) Comparison of tumor bioluminescence signals at different time points (*n* = 4). **J,** Histopathological analysis of mouse heart, liver, lung, kidney and brain in the CD19-CAR and GFD-CAR treatment groups. Scale bar, 50 μm. **K,** Serum AST and ALT levels in mice from CD19 CAR and GFD-CAR groups at 21 days (*n* = 4 for the CD19 CAR group; *n* = 3 for the GFD-CAR group). **L,** 21 days after inection of CAR T cells, sections of representative liver tissue samples were collected were analyzed by immunofluorescence to detect the expression levels of CD31 and uPAR in NSG mice (*n* = 3 mice per condition). Data is presented as mean ± SD and analyzed by one-way ANOVA followed by Tukey’s multiple comparisons test (**C**), analyzed by unpaired two-tailed Student’s t test with Welch’s correction (**F**) or two-way ANOVA followed by Sidak’s multiple comparisons test (**E, G-I)**, or unpaired two-tailed Student’s *t*-test with Welch’s correction (**K, L**) (**p* ≤ 0.05; ***p* ≤ 0.01; ****p* ≤ 0.001; ns, not significant).

**Supplementary Figure 9. mGFD-CD3 treatment alleviates systemic inflammation in aged mice.**

**A,** Histopathological analysis of mouse heart, liver, lung, kidney and brain were performed in naturally aged mice treated with mCD3 or mGFD-CD3 and recorded 6 months after the first infusion. Scale bar, 50 μm. **B,** ELISA analysis for TNF-α and IL-6 in blood samples from mice after treatment with mGFD-CD3 or mCD3 at 24h and 120h. (n=4 for each group). Data is presented as mean ± SD and analyzed by unpaired two-tailed Student’s t test with Welch’s correction (***p* ≤ 0.01; ns, not significant). **C,** ELISA analysis for IL-6, TNF-α and IL-1β in blood samples collected from mice after treatment with mGFD-CD3 or mCD3 at 21 days. (n=3 each group). **D,** Transcriptome sequencing analysis for expression levels of representative genes from endothelial-, erythrocyte-, hepatocyte-, cholangiocyte-, T cell-, NK-, Monocyte-, Macrophage-, DC-, pDC-, B cell-, Plasma- cell types and urokinase (Plaur) expression in liver tissue samples collected from mice treated with mCD3 or mGFD-CD3 on day 21. The gene expression levels of the control group were normalized and compared to those of the experimental group. (n=3). **E,** GO and KEGG enrichment pathways of mGFD-CD3 compared to mCD3 (n=3).

**Supplementary Figure 10. Behavioraland physiological characteristics of mGFD-CD3 treated mice.**

**A,** The speed, distance, hanging endurance and grip strength attributes of naturally aged mice treated with mCD3 or mGFD-CD3 recorded 21 days after the first infusion. (*n* = 5 for young group; *n* = 3 for mCD3 group; *n* = 3 for mGFD-CD3 group). **B,** Histopathological analysis of mouse muscle in naturally aged mice treated with mCD3 or mGFD-CD3 performed 21 days after the first infusion. (*n* = 5 for young group; *n* = 3 for mCD3 group; *n* = 3 for mGFD-CD3 group). Relative cell areas in the gastrocnemius were quantified (*left* panel) and analyzed by Image J analysis software. Scale bar, 50 μm. (ns, not significant). **C,** Representative images of immunofluorescence analysis performed at 24h post-injection of mGFD-CD3 to examine the colocalization F4/80 and uPAR in the liver and lung tissues of naturally aged mice (*n* = 3 for each time point). Macrophage cells are shown by the F4/80-positive area. **D,** Neutrophil- and lymphocyte counts were measured at serial time points after administration of mGFD-CD3 or mCD3 (*n* = 3 mice per condition). Data is presented as mean ± SD and analyzed by unpaired two-tailed Student’s t test with Welch’s correction(**A**-**C**). (***p ≤ 0.001; ns, not significant).

**Supplementary Figure 11. Treatment with mGFD-CD3 ameliorates age-associated phenotypes in naturally aged mice three months after the initial injection.**

**A,** Histopathological analysis of mouse heart, liver, lung, kidney and brain tissues were performed with naturally aged mice treated with mCD3 or mGFD-CD3, sampled 3 months after the first infusion (*n* = 5 for young group; *n* = 3 for mCD3 group; *n* = 3 for mGFD-CD3 group). Scale bar, 50 μm. **B,** Serum AST and ALT levels in mice were determined using a Biochemical Analyzer. **C,** Daily food consumption of each mouse. Data were normalized to mouse body weights. **D,** Weight changes of aged mice treated with mCD3 or mGFD-CD3 (*n* = 5 per condition). Results are representative of N = 2 independent experiments. **E,** Tracking animal movements using an open field test. Analyses included total distance moved, time spent moving, acceleration. **F,** (left) Representative images of H&E staining of IAT samples performed on naturally aged mice treated with mCD3 or mGFD-CD3, sampled 6 months after the first infusion. Quantification of relative adipocyte areas (normalized to the young mice) (*n*=5 mice per condition). Scale bar, 50 μm. **G**, (upper) Levels of glucose before (0 min) and after the intraperitoneal administration of insulin (0.5U/kg) (30, 60, 120 mins). (lower) Area under the curve (AUC) for the results from (g) (*n* = 5 for young group; n = 3 for mCD3 group; *n* = 3 for mGFD-CD3 group). The mouse experiments are representative results from two independent experiments. Data are presented as mean ± SD and analyzed by one-way ANOVA followed by Tukey’s multiple comparisons test (a-g) (*P ≤ 0.05; ns, no significant).

**Supplementary Figure 12. Liver function, hematological parameters and six accessible vital sign parameters are continuously monitored on Days 14, 42 and 90, after the first injection.**

**A,** Liver function was assessed on Days 14, 42 and 90. **B,** hematological parameters were assessed on Days 14, 42 and 90.**C,** Six accessible vital sign parameters were continuously monitored on Days 14, 42 and 90.

**Supplementary Figure 13. Gating strategies**

**A**. A gating strategy for CAR-T Positive. **B**, A gating strategy for BiTE binding to T cells. **C**, A gating strategy for BiTE binding to senescence cells. **D**, A gating strategy for BiTE binding to 293T cells overexpressing UPAR.
